# Absolute Configuration Determination with Electronically Enhanced Vibrational Circular Dichroism

**DOI:** 10.1002/anie.202517979

**Published:** 2025-11-17

**Authors:** Mariia Sapova, Chandan Kumar, Sahar Ashtari‐Jafari, Wybren J. Buma, Lucas Visscher

**Affiliations:** ^1^ Department of Chemistry and Pharmaceutical Sciences Faculty of Sciences Vrije Universiteit Amsterdam De Boelelaan 1108 Amsterdam 1081 HZ The Netherlands; ^2^ Van ’t Hoff Institute for Molecular Sciences University of Amsterdam Science Park 904 Amsterdam 1098 XH The Netherlands; ^3^ Institute for Molecules and Materials, FELIX Laboratory Radboud University Toernooiveld 7c Nijmegen 6525 ED The Netherlands

**Keywords:** Chirality, Circular dichroism, Quantum chemistry, Transition metals, Vibrational spectroscopy

## Abstract

Intensity enhancement in vibrational circular dichroism (VCD) arises in open‐shell transition metal complexes from coupling between ground‐state vibrational transitions and magnetic dipole‐allowed transitions to low‐lying excited states (LLESs). In this work, we apply Nafie's vibronic coupling theory to Me(II)‐(‐)‐sparteine‐${\mathrm{Cl}}_{2}$ (Me = Zn, Co, Ni) complexes to investigate these enhancement effects. We show that the VCD intensity is extremely sensitive to the excitation energies that neither time‐dependent density functional theory (TDDFT) nor state‐averaged complete active space self‐consistent field (SA‐CASSCF) calculations can predict with sufficient accuracy. We argue that instead of using more accurate quantum chemistry methods these excitation energies can be treated as parameters and optimized against experimental spectra. With this approach, we obtain simulated VCD similarity scores above 0.4, a threshold considered reliable for absolute configuration assignment. The ability to quantitatively reproduce enhanced experimental spectra with computations opens up new research areas, offering amongst else unique possibilities for the study of the chiral structure of systems such as transition metal complexes and metalloproteins that so far have remained intractable.

## Graphical abstract

We introduce a practical workflow for simulating vibrational circular dichroism spectra enhanced by vibronic coupling. We achieve qualitative agreement with experiment for Co(II) and Ni(II) spartein complexes, circumventing inaccuracies in theoretical excitation energy predictions. We show that even with economical density functional theory, high VCD similarity scores can be reached, paving the way to absolute configuration assignment.

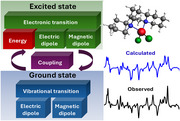

## Introduction

Natural products and other molecules of interest for drug discovery often contain one or more chiral centers that are crucial for their biological activity. This characteristic makes it essential to be able to determine their absolute configuration (AC) or enantiomeric composition.^[^ ^1^, ^2^, ^3^, ^4^, ^5^ ^]^ Electronic circular dichroism (ECD) spectroscopy is a spectroscopic method often employed to obtain insight, but its structural information content is relatively limited. Vibrational circular dichroism (VCD) offers in this respect significantly more information.^[^ ^6^ ^]^ VCD spectra are feature‐rich, provide detailed information of the chemical bonds close to the chiral centers, and give insight into intermolecular interactions.^[^ ^7^ ^]^ Many reviews cover both experimental and theoretical VCD methods,^[^ ^2^, ^3^, ^4^, ^8^, ^9^, ^10^ ^]^ but they typically focus on closed‐shell systems for which the electronic ground state is well separated from excited states. In those cases the VCD spectrum can be efficiently modeled using the magnetic field perturbation (MFP) theory of Stephens^[^ ^11^ ^]^ although the uncertainty in conformer energies can affect the reliability of the stereochemical assignment.^[^ ^12^ ^]^

For chiral molecules with low‐lying excited states (LLESs), as is typically the case in transition metal complexes, the situation is different. Such molecules are highly relevant in chiral material science and asymmetric catalysis^[^ ^13^, ^14^ ^]^ and there is therefore a growing interest in applying VCD spectroscopy to probe their chiral structure.^[^ ^15^, ^16^, ^17^, ^18^, ^19^ ^]^ Due to the presence of these low‐lying electronic states, the vibrational and electronic degrees of freedom of the molecule are not well separated, which has been found to lead to significant enhancements of VCD signals. The phenomenon of enhanced VCD signals in transition metal complexes has been known since 1980, when the first experimental VCD spectra for the sparteine complexes Me(II)‐(‐)(sparteine)${\mathrm{Cl}}_{2}$ (Me = Zn, Co, Ni) were reported in a narrow region of CH stretching modes.^[^ ^20^ ^]^ Later He et al. extended the measurement to the fingerprint region, where the open‐shell Co(II) and Ni(II) complexes have about ten times higher VCD intensity compared to the closed‐shell Zn complex.^[^ ^21^ ^]^ Pescitelli and Di Bari recently published a comprehensive review compiling available data on VCD enhancement and classifying the systems that exhibit this phenomenon.^[^ ^22^ ^]^ They combined the systems with an open‐shell metal center into a separate group, which contains mononuclear and multinuclear transition metal complexes, lanthanide complexes, and heme proteins. In most cases, the enhancement can be attributed to the coupling of vibrational and electronic excitations; however, other mechanisms can also come into play.^[^ ^22^ ^]^ Domingos et al. reported VCD spectra for ligands attached to paramagnetic Co(II)^[^ ^23^, ^24^ ^]^ for which enhancements up to two orders of magnitude were observed. They showed that isostructural Co(III) complexes do not exhibit this intensity amplification, providing experimental evidence that the observed enhancement originates from vibronic coupling with LLESs. In complexes with Schiff base ligands, a sign reversal effect was observed along with the enhanced VCD signals, and this phenomenon was also attributed to the presence of LLESs in a series of studies by Pescitelli.^[^ ^25^, ^26^, ^27^ ^]^ Enhancement due to LLESs has also been observed in Me(III) complexes: for example, Ru(III)(acac)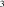 ^[^ ^28^ ^]^ and [Co(III)stien(biur)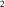 ${]\mathrm{NBu}}_{4}$.^[^ ^29^ ^]^

The enhanced intensity provides two practical benefits. First, the overall sensitivity of VCD improves significantly. Arrico et al., for example, applied this mechanism to enable a rapid and sensitive chirality recognition in α‐amino acids.^[^ ^30^ ^]^ Second, the enhancement is distance‐dependent, as was shown in the studies of Domingos et al. who demonstrated that amplification occurs locally within specific regions of the molecule, providing a way to “zoom in” on particular parts of a biomolecular system.^[^ ^31^ ^]^ However, in order to be able to use these potential applications, a reliable computational approach is needed to analyze and predict the observed enhanced VCD spectra. As yet, such an approach has been notoriously lacking.

Nafie developed the theoretical description of VCD for molecules with LLESs, employing a sum‐over‐states (SOS) formalism that incorporates coupling between vibrational normal modes and LLESs.^[^ ^32^ ^]^ Although Nafie's VCD vibronic theory^[^ ^32^ ^]^ provides a rigorous framework for vibronic enhancement, it has not been implemented in major quantum chemical software packages, and to date no ab initio calculations have been published that successfully reproduce enhanced VCD spectra. Tomeček and Bour^[^ ^33^ ^]^ suggested an alternative approach and studied Co(II)‐(‐)(sparteine)${\mathrm{Cl}}_{2}$ and bis[(S)‐N‐(1‐phenylethyl)salicylaldiminato]Δ‐Co(II) complexes (for brevity, we will from now on refer to these complexes as Co(II)(sp)${\mathrm{Cl}}_{2}$ and Co(II)(sal)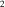, respectively). They employed a Herzberg–Teller expansion of the electronic wave function and incorporated nonadiabatic contributions in a model Hamiltonian, followed by full diagonalization of the Hamiltonian on the basis of adiabatic wave functions. Although their approach demonstrated clear enhancements, it was still unable to capture the correct signs for several VCD bands. In Co(II)(sp)${\mathrm{Cl}}_{2}$, for example, the calculated spectrum reproduced the overall band shape reasonably well, but the predicted enhancement remained roughly three times smaller than what was observed experimentally. Similarly, for Co(II)(sal)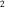, the characteristic monosignate VCD profile observed in the experiment could not be reproduced by the calculations.

In this work, we adopt Nafie's vibronic coupling theory of VCD and argue that previous attempts to predict enhanced VCD failed because existing quantum chemistry methods cannot describe low‐lying excited states with the required accuracy. Moreover, we discuss that the commonly accepted assumption that the potential energy surfaces of the LLESs do not differ significantly from that of the electronic ground state may not always hold and can influence significantly the predicted spectrum. We propose a computational protocol to address these limitations and demonstrate its effectiveness for Me(II)(sp)${\mathrm{Cl}}_{2}$ complexes. We therein show that—despite the uncertainties in excitation energy prediction by state‐of‐the‐art quantum chemical methods and neglect of Franck–Condon effects—a reliable identification of absolute chirality is possible. This opens the way for application of VCD as a sensitive tool for structure determination of a much broader range of compounds than has been possible with MFP.

## Results and Discussion

### Magnetic Field Perturbation Theory (MFP)

In Stephens MFP formalism for VCD,^[^ ^11^ ^]^ the VCD rotational strength $R_{i}$ is written as^[^ ^11^, ^34^, ^35^ ^]^

(1) $$R_{i}=Im\left[E_{i}^{tot}\cdot {M_{i}}^{tot}\right],$$

where i is the ith normal mode, and $E^{tot}$ and $M^{tot}$ are the total electric and magnetic dipole transition moments, respectively. The $E^{tot}$ and $M^{tot}$ vectors are defined as

(2) $$E_{\beta ,i}^{tot}={\left(\frac{\hbar }{\omega_{i}}\right)}^{\frac{1}{2}}\sum_{\lambda \alpha }P_{\alpha \beta }^{\lambda }S_{\lambda \alpha ,i}$$

(3) $$M_{\beta ,i}^{tot}=-{\left(2\hbar^{3}\omega_{i}\right)}^{\frac{1}{2}}\sum_{\lambda \alpha }A_{\alpha \beta }^{\lambda }S_{\lambda \alpha ,i},$$

where S is the transformation matrix from Cartesian (λα) to normal (i) nuclear coordinates, and P and A are the atomic polar tensor (APT) and the atomic axial tensor (AAT), respectively. $\omega_{i}$ is the harmonic angular frequency of the ith mode, λ is used to enumerate the nuclei and α and β indicate Cartesian components of either the nuclear displacement vectors or the electromagnetic field, respectively. The APT and AAT tensors can be written as a sum of electronic and nuclear contributions. The APT tensor is then given by

(4) $$P_{\alpha \beta }^{\lambda }=E_{\alpha \beta }^{\lambda }+N_{\alpha \beta }^{\lambda }$$

with the electronic contribution given by

(5) $$E_{\alpha \beta }^{\lambda }={\frac{\partial \langle \Psi_{g}\left(R\right)|\mu_{\beta }|\Psi_{g}\left(R\right)\rangle }{\partial R_{\lambda \alpha }}|}_{R=R_{0}}$$

and the nuclear contribution by

(6) $$N_{\alpha \beta }^{\lambda }=eZ_{\lambda }\delta_{\alpha \beta }.$$

The AAT tensor is given by

(7) $$A_{\alpha \beta }^{\lambda }=I_{\alpha \beta }^{\lambda }+J_{\alpha \beta }^{\lambda },$$

where $I^{\lambda }$ is the electronic contribution and $J^{\lambda }$ the nuclear contribution. The electronic contribution is given as the overlap of the perturbed wave functions with respect to nuclear displacements and with respect to magnetic field

(8) $$I_{\alpha \beta }^{\lambda }={〈\frac{\partial \Psi (R,B)}{\partial R_{\lambda \alpha }}|\frac{\partial \Psi (R,B)}{\partial B_{\beta }}〉|}_{R=R_{0},B=0}$$

with a nuclear contribution given by

(9) $$J_{\alpha \beta }^{\lambda }=i\frac{eZ_{\lambda }}{4\hbar c}\sum_{\gamma }\varepsilon_{\alpha \beta \gamma }R_{\lambda \gamma }^{0},$$

where $\varepsilon_{\alpha \beta \gamma }$ is the Levi–Civita symbol.

### Nafie's Vibronic Theory of VCD with LLESs

Stephens' efficient MFP formalism has been implemented in many quantum chemistry programs^[^ ^5^, ^11^, ^34^ ^]^ but does not account for vibronic coupling with LLESs. Equations to express this coupling in terms of additional LLESs contributions to the APT and the AAT tensors have been derived by Nafie^[^ ^32^ ^]^ and will be employed here. We refer to this paper for the full derivation and will only briefly discuss the SOS formulation of the APT and the AAT tensors.

In Nafie's SOS formulation of vibronic coupling, the APT tensor ($E_{\alpha \beta }^{A}\left(\omega_{a}\right)$) is written as

(10) $$E_{\alpha \beta }^{\lambda }\left(\omega_{a}\right)=2\sum_{e\neq g}\left[\frac{\omega_{eg}^{2}}{\omega_{eg}^{2}-\omega_{a}^{2}}\right]\langle \Psi_{g}|\mu_{\beta }|\Psi_{e}\rangle \langle \Psi_{e}|\frac{\partial \Psi_{g}}{\partial R_{\lambda \alpha }}\rangle {|}_{R=R_{0}}.$$

On the RHS of Equation (10), $\Psi_{g}$ is the ground state electronic wave function and $\Psi_{e}$ refers to electronically‐excited wave functions. The frequency corresponding to the vertical electronic excitation energy of state e is denoted as $\omega_{eg}$ while the normal mode frequency is given as $\omega_{a}$. In this formalism, the APT is dependent on the normal mode frequency, but it can be related to the one defined by Stephens by writing Equation (10) in terms of two contributions,

(11) $$\begin{array}{cc} E_{\alpha \beta }^{\lambda }\left(\omega_{a}\right) & =2\sum_{e\neq g}\left[1+\frac{\omega_{a}^{2}}{\omega_{eg}^{2}-\omega_{a}^{2}}\right]\langle \Psi_{g}|\mu_{\beta }|\Psi_{e}\rangle \langle \Psi_{e}|\frac{\partial \Psi_{g}}{\partial R_{\lambda \alpha }}\rangle {|}_{R=R_{0}}\\ & =2\sum_{e\neq g}\langle \Psi_{g}|\mu_{\beta }|\Psi_{e}\rangle \langle \Psi_{e}|\frac{\partial \Psi_{g}}{\partial R_{\lambda \alpha }}\rangle {|}_{R=R_{0}}\\ & +\underset{enhancementterm}{\underset{︸}{2\sum_{e\neq g}\frac{\omega_{a}^{2}}{\omega_{eg}^{2}-\omega_{a}^{2}}\langle \Psi_{g}|\mu_{\beta }|\Psi_{e}\rangle \langle \Psi_{e}|\frac{\partial \Psi_{g}}{\partial R_{\lambda \alpha }}\rangle {|}_{R=R_{0}}}}. \end{array}$$

The first term on the RHS of Equation (11) is equivalent to the APT defined in the MFP formalism,

(12) $$\begin{array}{cc} & 2\sum_{e\neq g}\langle \Psi_{g}|\mu_{\beta }|\Psi_{e}\rangle \langle \Psi_{e}|\frac{\partial \Psi_{g}}{\partial R_{\lambda \alpha }}\rangle {|}_{R=R_{0}}\\ & =2\langle \Psi_{g}|\mu_{\beta }|\frac{\partial \Psi_{g}}{\partial R_{A\alpha }}\rangle {|}_{R=R_{0}}-2\langle \Psi_{g}|\mu_{\beta }|\Psi_{g}\rangle \langle \Psi_{g}|\frac{\partial \Psi_{g}}{\partial R_{\lambda \alpha }}\rangle {|}_{R=R_{0}}\\ & ={\frac{\partial \langle \Psi_{g}|\mu_{\beta }|\Psi_{g}\rangle }{\partial R_{\lambda \alpha }}|}_{R=R_{0}}, \end{array}$$

since $\langle \Psi_{g}|\frac{\partial \Psi_{g}}{\partial R_{\lambda \alpha }}\rangle =0$ and the electron dipole moment operator carries no direct dependence on nuclear coordinates. The enhancement can thus be solely attributed to the second term of Equation (11), which depends both on the normal mode frequency and the electronic excitation energy.

Like the APT, the AAT in the SOS formalism depends on both the normal mode frequency and the electronic excitation energy. It takes the form

(13) $$\begin{array}{cc} I_{\alpha \beta }^{\lambda }\left(\omega_{a}\right) & =\sum_{e\neq g}\left[\frac{\omega_{eg}^{2}}{\omega_{eg}^{2}-\omega_{a}^{2}}\right]\frac{\left\langle \frac{\partial \Psi_{g}}{\partial R_{\lambda \alpha }}|\Psi_{e}\right\rangle \langle \Psi_{e}|m_{\beta }\left|\Psi_{g}\right\rangle }{\omega_{eg}}{|}_{R=R_{0}}, \end{array}$$

where m is the magnetic dipole operator and we have multiplied the original definition of Nafie by a factor of $\frac{i}{2}$ to be consistent with the one of Stephens.^[^ ^8^ ^]^ Equation (13) can be partitioned as

(14) $$\begin{array}{cc} I_{\alpha \beta }^{A}\left(\omega_{a}\right) & =\sum_{e\neq g}\left[1+\frac{\omega_{a}^{2}}{\omega_{eg}^{2}-\omega_{a}^{2}}\right]\frac{\left\langle \frac{\partial \Psi_{g}}{\partial R_{A\alpha }}|\Psi_{e}\right\rangle \langle \Psi_{e}|m_{\beta }\left|\Psi_{g}\right\rangle }{\omega_{eg}}{|}_{R=R_{0}}\\ & =\sum_{e\neq g}\frac{\left\langle \frac{\partial \Psi_{g}}{\partial R_{A\alpha }}|\Psi_{e}\right\rangle \langle \Psi_{e}|m_{\beta }\left|\Psi_{g}\right\rangle }{\omega_{eg}}{|}_{R=R_{0}}\\ & +\underset{enhancementterm}{\underset{︸}{\sum_{e\neq g}\left[\frac{\omega_{a}^{2}}{\omega_{eg}^{2}-\omega_{a}^{2}}\right]\frac{\left\langle \frac{\partial \Psi_{g}}{\partial R_{A\alpha }}|\Psi_{e}\right\rangle \langle \Psi_{e}|m_{\beta }\left|\Psi_{g}\right\rangle }{\omega_{eg}}{|}_{R=R_{0}}}}. \end{array}$$

The first term of Equation (14) can be related to Equation (8):

(15) $$\begin{array}{cc} I_{\alpha \beta }^{A} & ={〈\frac{\partial \Psi_{g}}{\partial R_{A\alpha }}|\frac{\partial \Psi_{g}}{\partial B_{\beta }}〉|}_{R=R_{0},B=0}\\ & =\sum_{e\neq g}\frac{\langle \frac{\partial \Psi_{g}}{\partial R_{A\alpha }}|\Psi_{e}\rangle \langle \Psi_{e}|m_{\beta }\left|\Psi_{g}\right\rangle }{\omega_{eg}}{|}_{R=R_{0}}, \end{array}$$

while the second term of Equation (14) represents the enhancement.

These equivalences make it possible to compute the VCD rotational strength as a sum of standard and enhancement contributions and thereby allow for detailed analysis. We define

(16) $$\begin{array}{cc} & R_{i}^{enh}=Im\left[E_{i}^{tot}\cdot M_{i}^{enh}+E_{i}^{enh}\cdot M_{i}^{tot}\right]\\ & E_{i,\beta }^{enh}=2{\left(\frac{\hbar }{\omega_{i}}\right)}^{\frac{1}{2}}\sum_{e\neq g}\frac{\omega_{i}^{2}}{\omega_{eg}^{2}-\omega_{i}^{2}}{[\langle \Psi_{g}|\mu_{\beta }\left|\Psi_{e}\right\rangle \left\langle \Psi_{e}|\frac{\partial \Psi_{g}}{\partial R_{i}}\right\rangle ]}_{R=R_{0}}\\ & M_{i,\beta }^{enh}=-{\left(2\hbar^{3}\omega_{i}\right)}^{\frac{1}{2}}\sum_{e\neq g}\frac{\omega_{i}^{2}}{\omega_{eg}^{2}-\omega_{i}^{2}}[\frac{\left\langle \Psi_{g}|m_{\beta }|\Psi_{e}\right\rangle }{\omega_{eg}}\left\langle \Psi_{e}\right|{\frac{\partial \Psi_{g}}{\partial R_{i}}\rangle ]}_{R=R_{0}}. \end{array}$$

The enhancement corrections can be calculated separately and require evaluation of excitation energies, electric and magnetic dipole transition moments for the LLESs as are readily available in most quantum chemistry codes. The required non‐adiabatic couplings (NACs) $\left\langle \Psi_{e}|\frac{\partial \Psi_{g}}{\partial R_{A\alpha }}\right\rangle$ can also be obtained from a number of programs.

Nafie's formulation employs the vertical approximation in which the ground and excited state potential energy surfaces are assumed to have the same shape and to differ only by a constant energy shift. Within this approximation, one has $\phi_{ev}^{a}=\phi_{gv}^{a}$ for all vibrational states (labeled v) of normal mode (a) of the electronic ground (g) and excited states (e). Consequently, overlap integrals of the form $\left\langle \phi_{g0}^{a}|\phi_{ev}^{a}\right\rangle$ are zero for all transitions except the fundamental 0→0 transition. As we show in the results section, however, this vertical approximation does not hold for all low‐lying excited states of sparteine complexes and is one of the reasons for previously observed differences between calculated and observed spectra.

### Sparteine Complex

**Figure 1:**
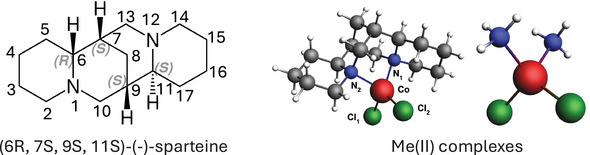
Structural formula of (6R, 7S, 9S, 11S)‐(‐)‐sparteine ligand (left). Co(II)(sp)${\mathrm{Cl}}_{2}$ structure optimized on BP86‐D3BJ/def2‐TZVP level with CPCM treatment of ${\mathrm{CHCl}}_{3}$ solvent effects and the $C_{2v}$ Co(II)(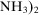 ${\mathrm{Cl}}_{2}$ model structure.

We study the effects of low‐lying excited states on the VCD spectra of the Me(II)(sp)${\mathrm{Cl}}_{2}$ (Me = Co, Ni) open‐shell complexes. The cobalt ($d^{7}$ electronic configuration) and nickel ($d^{8}$ electronic configuration) atoms form high‐spin open‐shell, four‐coordinate complexes with the bidentate chiral (‐)‐sparteine ligand with the absolute configuration of 6R, 7S, 9S, 11S (Figure 1). They can be compared with analogous zinc complexes where the electronic structure is that of a closed‐shell configuration without unpaired spins with significantly higher excitation energies, and therefore negligible enhancement effects. We validated the ground state computational scheme on Zn(II)(sp)${\mathrm{Cl}}_{2}$ before applying it for Co(II) and Ni(II) complexes (see Computational Details in the Supporting Information). To gain more insight about the electronic structure of the excited states, we also study model complexes with $C_{2v}$ symmetry. These model complexes are constructed by truncating and symmetrizing the optimized sparteine complexes while preserving bond distances around the transition metal.

Enhanced VCD spectra were computed using our Python implementation^[^ ^36^ ^]^ of Nafie's vibronic coupling theory. To assess agreement with the experiment, we used the simIR and simVCD metrics from Shen et al.^[^ ^37^ ^]^ Experimental spectra were digitized from the thesis of He^[^ ^38^ ^]^ using a Python script.

### Excited State Calculations

We investigate with TDDFT and SA‐CASSCF the excitation energies and nature of LLESs for the Co(II)(sp)${\mathrm{Cl}}_{2}$ and Ni(II)(sp)${\mathrm{Cl}}_{2}$ complexes. The contributions of each excited state to the enhanced VCD spectra depend on the EDTM, MDTM, excitation energy, non‐adiabatic coupling matrix elements, and vibrational frequencies, so the quality of all these quantities is important to obtain a reliable estimate of the enhancement.

**Figure 2:**
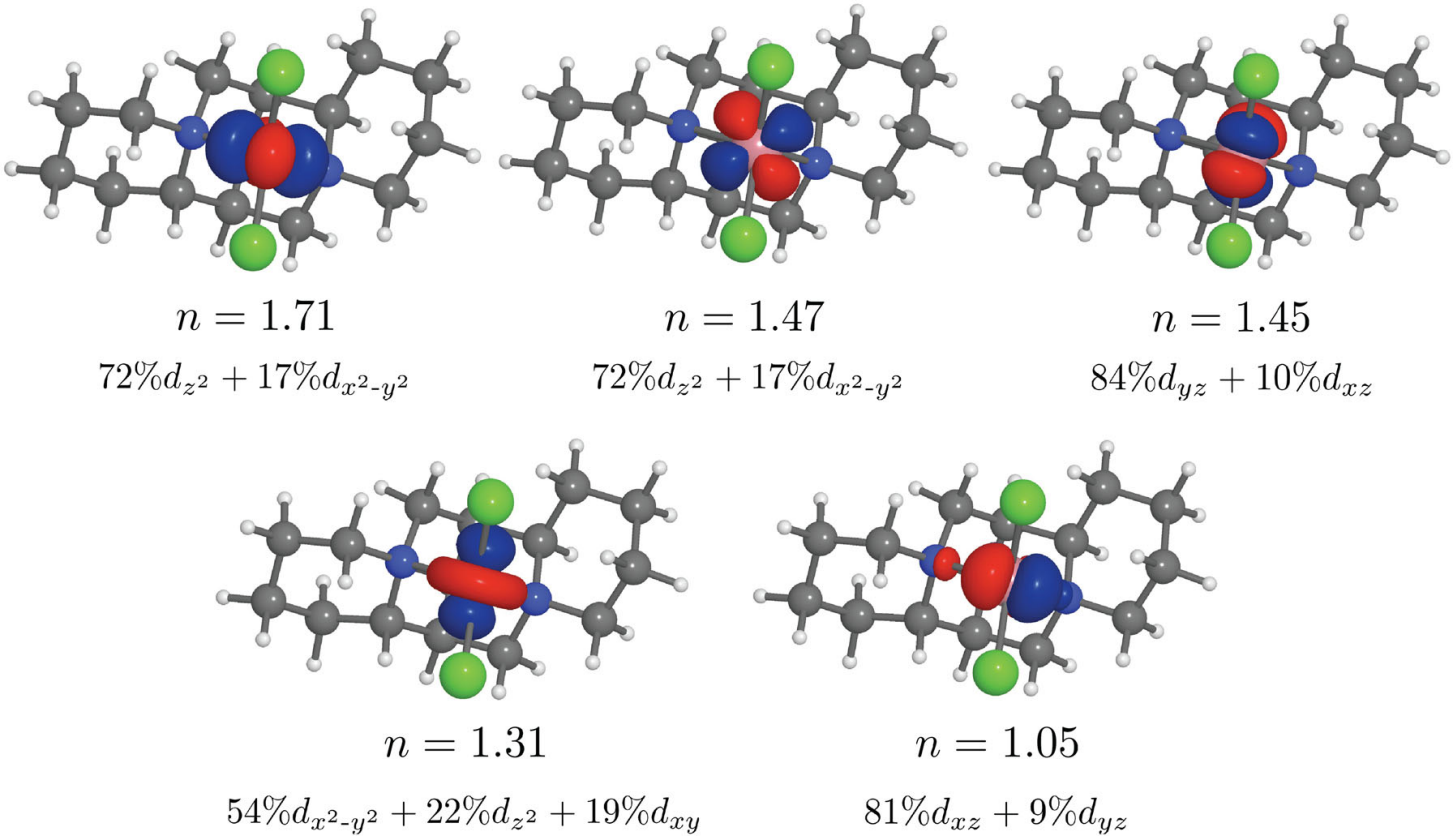
SA‐CASSCF(7, 5) natural active orbitals of Co(II)(sp)${\mathrm{Cl}}_{2}$ with occupation numbers (n). Löwdin orbital compositions are shown for atomic orbitals contributing more than 5%.

According to experimental studies, pseudotetrahedral Me(II)(sp)${\mathrm{Cl}}_{2}$ complexes exhibit low‐lying, electric dipole‐forbidden d–d transitions that underlie their enhancement effect.^[^ ^21^ ^]^ Our SA‐CASSCF calculations confirm the dominant d character of active orbitals with Löwdin orbital compositions 94% and higher (see Figure 2 for Co(II), Table S7 for Ni(II)). The localized character of the LLESs character is confirmed by larger SA‐CASSCF calculations (Tables S2, S3, S6, and S8). Since the transitions of interest are highly localized, we also construct a smaller model complex Me(II)(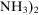 ${\mathrm{Cl}}_{2}$ with $C_{2v}$ symmetry. This higher symmetry allows state‐specific CASSCF calculations, thereby avoiding possible artifacts introduced by orbital averaging in SA‐CASSCF.

**Figure 3:**
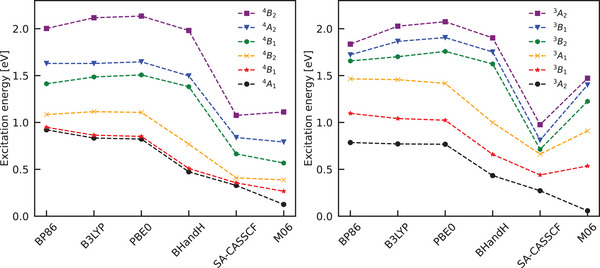
Excitation energies of the first six excited states of Co(II)(sp)${\mathrm{Cl}}_{2}$ (left) and Ni(II)(sp)${\mathrm{Cl}}_{2}$ (right) calculated with TDDFT and SA‐CASSCF in Orca.^[^ ^40^ ^]^ To distinguish the excited states, we use for non‐symmetric sparteine the symmetry labels of the $C_{2v}$ model complex (see Table 1). The functionals are ordered from GGA (BP86) to hybrid functionals with increasing Hartree‐Fock exchange: B3LYP (20%), PBE0 (20%), BHandH (50%). The Hybrid Meta‐GGA functional M06 yields a too low first excitation energy and is put to the right of the reference results to facilitate the comparison.

Co(II)(sp)${\mathrm{Cl}}_{2}$ SA‐CASSCF(7, 5) calculations show that the first three excited states lie approximately 0.2 eV below the next higher state (see Figure 3). Since the contribution of each state scales inversely with $w_{eg}-w_{a}$, only excited states with energies that nearly coincide with the frequency of a vibrational mode yield significant enhancements. Contributions from the fourth and higher excited states are thus, for all practical purposes, negligible. For Ni(II)(sp)${\mathrm{Cl}}_{2}$, we find on the other hand that there is already a significant energy gap between the first and second excited state (see Figure 3), and for this complex, we therefore only need to consider the first excited state in the enhancement calculations. To evaluate to what extent excitation energies calculated at the TDDFT level are able to reproduce SA‐CASSCF result, we compare them in Figure 3 with representative GGA, hybrid, and hybrid meta‐GGA functionals. From this figure, it becomes clear that pure GGAs substantially overestimate the energies of the low‐lying states. Introducing Hartree–Fock exchange systematically improves the agreement: B3LYP (20% HF exchange) reduces the error, and BHandH (50% HF exchange) performs even better. In contrast, the hybrid meta‐GGA M06^[^ ^39^ ^]^ yields a first excitation energy that is too low relative to the SA‐CASSCF benchmark values.

In order to determine whether the use of state‐averaged orbitals for the sparteine complexes does not introduce mixing of the character of the electronic states, we compare in Table 1 CASSCF/NEVPT2 excitation energies for the sparteine and symmetric model complexes. Notably, the SA‐CASSCF(7,5) results for Co(II)(sp)${\mathrm{Cl}}_{2}$ and Co(II)(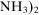 ${\mathrm{Cl}}_{2}$ are nearly identical, confirming the highly localized character of these transitions. For the $C_{2v}$ complex the ground (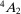) and excited states (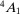, 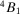 and 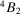) belong each to different irreducible representations, which permits state‐specific CASSCF optimizations. Here, the first two excited states are almost degenerate, while the third state lies slightly higher in energy. For the Ni(II)(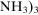 ${\mathrm{Cl}}_{2}$ complex, the ground state is 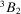, while the three lowest excited states are the 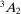, 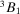, and 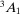 states. Overall, the close agreement between the SS‐CASSCF energies of the high‐symmetry model and the SA‐CASSCF results confirms that the state‐averaged orbitals provide a reliable description of these local excitations.

**Table 1:** Vertical (v) and adiabatic (a) excitation energies (eV) of (‐)‐sparteine and model complexes calculated at the CASSCF level. NEVPT2 corrected energies are given in brackets.

	Co(II)( 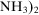 ${\mathrm{Cl}}_{2}$		Co(II)(sp)${\mathrm{Cl}}_{2}$	
Symmetry	SS‐CASSCF (**v**) (NEVPT2)	SA‐CASSCF (**v**) (NEVPT2)	SA‐CASSCF (**v**) (NEVPT2)	SA‐CASSCF (**a**) (NEVPT2)
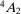 → 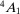	0.35 (0.39)	0.34 (0.44)	0.34 (0.42)	0.22 (0.34)
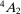 → 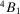	0.34 (0.42)	0.37 (0.45)	0.37 (0.48)	0.32 (0.51)
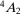 → 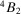	0.41 (0.50)	0.41 (0.52)	0.42 (0.53)	0.26 (0.45)

	Ni(II)( 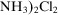		Ni(II)(sp)${\text{Cl}}_{2}$	
Symmetry	SS‐CASSCF (**v**) (NEVPT2)	SA‐CASSCF (**v**) (NEVPT2)	SA‐CASSCF (**v**) (NEVPT2)	SA‐CASSCF (**a**) (NEVPT2)
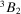 → 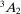	0.30 (0.35)	0.29 (0.37)	0.30 (0.39)	0.25 (0.34)
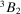 → 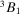	0.48 (0.52)	0.43 (0.57)	0.47 (0.61)	0.45 (0.72)
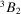 → 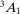	0.63 (0.72)	0.62 (0.83)	0.70 (0.92)	0.53 (0.91)

As discussed above, one of the key approximations in the SOS formalism is that the potential energy surfaces of ground and excited states are not displaced with respect to each other. To check whether this assumption holds for the present systems, we show in the last column of Table 1 the adiabatic excitation energies of the Co(II) and Ni(II) spartein complexes. For the ground state, we retain the BP86 geometry as it yields geometries in best agreement with experimental data (see Figures S1–S4). For excited‐state geometry optimizations, we used the BHandH functional, as it better mimics the SA‐CASSCF state description than BP86 (see Table S4). Since TDDFT gradient calculations share terms with non‐adiabatic couplings (NACs), a functional yielding more accurate NACs is likely to provide a more reliable description of excited‐state gradients. For Co(II)(sp)${\mathrm{Cl}}_{2}$, we find that geometry relaxation of the first and the third excited state lowers the excitation energy by more than 0.10 eV, which is more than a third of the vertical excitation energy and thus quite significant. For the second excited state, the relaxation energy is much smaller, although still more than 10% of its vertical excitation energy. For Ni(II)(sp)${\mathrm{Cl}}_{2}$, similar observations are made, although in this case the relaxation energy of the first excited state is somewhat lower.

The important conclusion that we thus need to draw is that applying the vertical approximation within the SOS formalism will have a significant impact on the outcome of the calculations.^1^ To achieve quantitatively accurate enhanced VCD predictions by fully ab initio calculations would require explicit calculations of vibrational overlap integrals and their inclusion in the calculation. This will lead, however, to impractically large computational demands, and alternative solutions are needed. In the next sections we will therefore introduce a readily usable semi‐empirical approach and demonstrate that such an approach allows for calculations that quantitatively reproduce experimental VCD spectra.

### SA‐CASSCF Calculations of Enhancements

**Figure 4:**
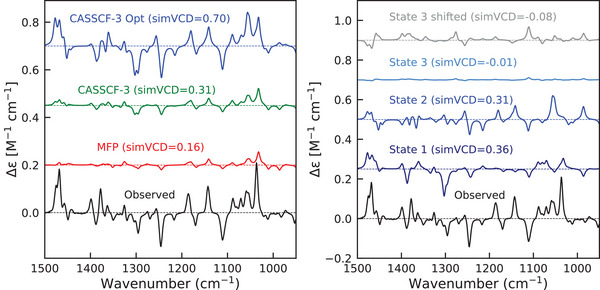
Left panel: enhanced VCD spectra of Co(II)(sp)${\mathrm{Cl}}_{2}$ calculated using MFP (red), SA‐CASSCF (green), and SA‐CASSCF with optimized excited state energies (blue). The number 3 indicates that 3 states are included in the optimization. **Right panel**: Analysis of the optimization result by plotting individual contributions of the excited states. The first two states both have excitation energies 0.23 of eV and give rise to a simVCD value of 0.36 (dark blue) and 0.31 (blue), respectively. The contribution of the third excited state is almost zero (light blue). The grey line corresponds to the excitation energy of the third state shifted to 0.23 eV in which case a simVCD value of ‐0.08 is obtained.

We compute enhanced VCD spectra using our Python implementation of Equation (16). To this purpose, the APT and AAT tensors are evaluated at the BP86‐D3BJ/CPCM level, and corrections to these tensors are obtained from OpenMolcas SA‐CASSCF calculations averaged over four states (see Computational Details in Supporting Information). The results of VCD calculations for Co(II)(sp)${\mathrm{Cl}}_{2}$ are shown in the left panel of Figure 4. Inclusion of three excited states with unmodified vertical excitation energies slightly improves the simVCD value over MFP calculation, but fails to reproduce the pronounced experimental enhancement. Given the uncertainties in the computed excitation energies and the limitations of the vertical approximation, we treat the excited‐state energies as tunable parameters, optimizing them to maximize simVCD.

This follows our previous approach for handling uncertainty in Boltzmann weights during conformational averaging.^[^ ^41^ ^]^ We thus take the transition dipole moments and non‐adiabatic couplings directly from the SA‐CASSCF calculations, modifying only the energies of the three lowest excited states. Using this approach, we can achieve very good agreement with the experimental spectrum of Co(II)(sp)${\mathrm{Cl}}_{2}$ with a simVCD value of 0.70. The energies of the first two excited states converge to a value of 0.23 eV, which is approximately 0.1 eV lower than the value obtained with SA‐CASSCF calculations. Such a difference is quite acceptable and is well within the expected error of this method. We find that the energy of the third state reaches a preset upper bound value of 1 eV, indicating that it does not contribute to the enhancement. This is caused by the negative similarity between the third state contribution and the observed spectra, which makes the optimization procedure raise its energy from the initially estimated 0.42 eV. Also, at 0.42 eV, its contribution is negligible, this reduces the number of tunable parameters in our procedure to just two.

**Figure 5:**
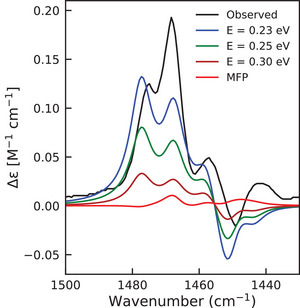
Co(II)(sp)${\mathrm{Cl}}_{2}$ VCD intensity dependence on the excitation energies of the first two excited states in the 1430–1500 ${\mathrm{cm}}^{-1}$ region.

The computed VCD enhancement is highly sensitive to the lowest two excitation energies (Figure 5). The figure demonstrates that even a small 0.02 eV shift in excitation energy dramatically amplifies the bands. The calculated bands are slightly shifted relative to the experimental spectrum because we applied only a single frequency scaling factor from the IR calculations. Such a small energy difference far exceeds the typical uncertainty of even the highest‐level quantum chemistry methods available. Consequently, fitting low‐lying excitation energies to experimental VCD data would be more than justified and is a quite practical route to reproduce the observed enhancements.

The individual contributions of each excited state to the enhanced VCD spectrum are shown on the right of Figure 4. Using only the first or only the second excited state yields 0.36 and 0.31 similarity with the observed spectra (without considering MFP part). The third excited state gives virtually no similarity to the observed spectrum indicating that its energy lies above the resonance window required for enhancement. When artificially shifted to 0.23 eV (gray trace), the third state has a negative simVCD value. The enhancements observed in the experimental spectrum thus actually provide a fingerprint of the extent to which each excited state is of importance in determining the enhancement of that band.

**Figure 6:**
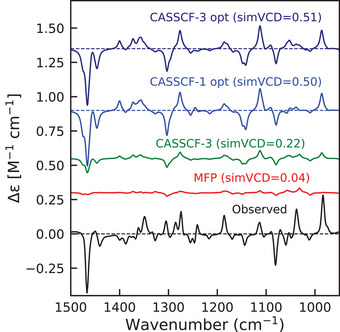
Enhanced VCD spectra of Ni(II)(sp)${\mathrm{Cl}}_{2}$ calculated with MFP (red, simVCD = 0.04), SA‐CASSCF (green, simVCD = 0.22), and SA‐CASSCF with optimized energies, including one (blue, simVCD = 0.50) and three states (dark blue, simVCD = 0.51).

For Ni(II)(sp)${\mathrm{Cl}}_{2}$, the enhanced spectra with unmodified SA‐CASSCF energies also show only a slight enhancement compared to MFP spectra ( Figure 6). Optimization of the excitation energies of the three lowest excited states gives a simVCD value of 0.51 with optimized energies of 0.24 and 0.30 eV for the first two states, while for the third state the optimization pushes the excitation energy to the upper bound of 1.0 eV. Optimizing with only the lowest excited state yields virtually identical spectra, however, indicating that in this case a single state drives the enhancement (see Figure S7). This agrees with the SA‐CASSCF results, which predict that the second and third excited states lie significantly higher than the first one. After optimization, the calculated Ni(II)(sp)${\mathrm{Cl}}_{2}$ spectrum shows as most notable discrepancy an intense negative peak near 1300 ${\mathrm{cm}}^{-1}$, which is absent in the experimental spectrum. This feature also appears in the MFP spectra and might be due to artifacts arising from the implicit modeling of solvent effects. Other discrepancies are seen in the 1000–1200 region, but this region is rather crowded with many vibrations contributing and is therefore difficult to model quantitatively correctly. If we exclude only the 1300–1320 ${\mathrm{cm}}^{-1}$ region, a simVCD value of 0.57 is obtained. Irrespective of whether this region is included or not, the overall conclusion remains, however, that our approach enables a reliable assignment of the absolute configuration of the complex.

We conclude that by tuning the lowest excitation energies, SA‐CASSCF is able to predict VCD spectra of sparteine complexes that are in good agreement with the experimental spectra. However, SA‐CASSCF calculations of non‐adiabatic couplings will present a serious bottleneck for applying this approach to larger systems. To overcome this computational bottleneck, we will next demonstrate that TDDFT can be employed as a more scalable alternative to model VCD intensity enhancements.

### TDDFT Calculations of Enhancements

**Figure 7:**
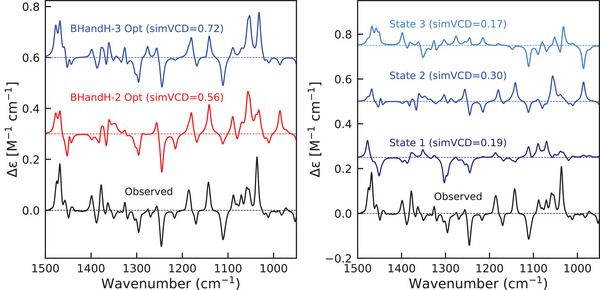
Left panel: Enhanced VCD spectra of Co(II)(sp)${\mathrm{Cl}}_{2}$ calculated with the BHandH functional, including two (red, simVCD = 0.56) and three excited states (blue, simVC = 0.72). Right panel: Individual contributions of the first three excited states to the enhancements in the VCD spectrum of Co(II)(sp)${\mathrm{Cl}}_{2}$ using BHandH leading to simVCD values of 0.19, 0.30, and 0.17, respectively.

Most density functional approximations (DFAs) that we studied predict excitation energies that are too high to contribute to the enhancements (see Tables S9 and S10). To remedy this, we optimize the excitation energies in the same way as in the SA‐CASSCF approach, while retaining the transition dipole moments and non‐adiabatic couplings from the TDDFT calculations. We find that calculations using BHandH and B3LYP yield nearly identical simVCD values, and we therefore report here the BHandH results (see Figure 7). Data for B3LYP and other DFAs is available in the Supporting Information.

**Figure 8:**
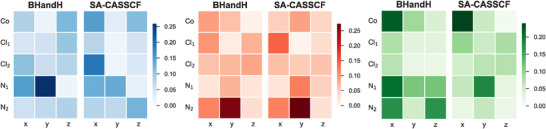
Non‐adiabatic couplings between the ground and first three excited states of Co(II)(sp)${\mathrm{Cl}}_{2}$ calculated with BHandH and SA‐CASSCF for atoms around Co(II): (left) first excited state; (middle) second excited state; (right) third excited state.

For Co(II)(sp)${\mathrm{Cl}}_{2}$, optimizing the energies of the three lowest excited states with BHandH brings all of them to around 0.23 eV. This differs from the SA‐CASSCF results, where the third state remains higher and does not contribute to the enhancement. We attribute this to differences in non‐adiabatic couplings (NACs) (see Figure 8). The only significant coupling values correspond to atoms directly bonded to the transition metal, confirming the distance‐dependence of the enhancement effect. Comparison of the BHandH and SA‐CASSCF NACs between the ground and second excited states shows that they are quite similar. This is not the case for the first and third excited state. In particular we notice that NACs between the ground and third excited state involving the N atoms are noticeably stronger with BHandH, which likely enables its contribution to the enhancement. B3LYP yields NACs closely matching those from BHandH, explaining the nearly identical enhanced VCD profiles. NACs and their mean errors for other DFAs can be found in the Supporting Information.

Restricting the optimization to just two lowest excited states still yields good agreement with experiment. The simVCD value is reduced to 0.56 compared to 0.72 when all three states are adjusted—which is still acceptable for reliable assignment of the absolute configuration—but equally important is that the overall spectral profile is still well reproduced. Most of the discrepancy arises in the 1250–1400 ${\mathrm{cm}}^{-1}$ region, and from the more pronounced negative peak around 1100 ${\mathrm{cm}}^{-1}$. The right hand panel of Figure 7 shows the contributions of each excited state, again illustrating that each state has a unique enhancement fingerprint both in sign and magnitude. For instance, the first excited state produces a negative feature near 1300 ${\mathrm{cm}}^{-1}$, while both the second and third excited states give positive peaks at that frequency.

**Figure 9:**
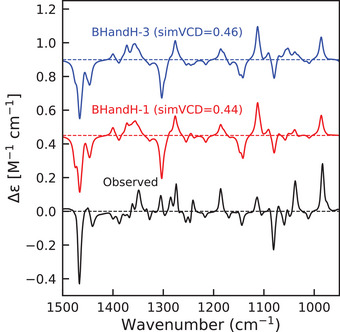
Enhanced VCD spectra of Ni(II)(sp)${\mathrm{Cl}}_{2}$ calculated with BHandH functional with optimized energies, including one (red) and three states (blue).

For Ni(II)(sp)${\mathrm{Cl}}_{2}$, the TDDFT results using the BHandH functional are essentially identical to those obtained with SA‐CASSCF: only the first excited state contributes to the enhancement, giving a final simVCD value of 0.44 (see Figure 9) and an optimized energy of 0.23 eV. The NACs computed with BHandH closely match those from SA‐CASSCF for Ni(II)(sp)${\mathrm{Cl}}_{2}$ (see Figure S15), which explains that both approaches lead to the same result and confirms our explanation for the differences found for the Co complex. Similar to what was found with SA‐CASSCF, we also observe with TDDFT a large negative peak at 1300 ${\mathrm{cm}}^{-1}$.

### Assignment of Absolute Configuration

Because the excitation energies are adjusted via an optimization procedure, overfitting is a natural concern. To critically evaluate our approach, we test its ability to provide a reliable assignment of the absolute configuration. To probe it we invert the experimental Me(II)‐(‐)‐sparteine‐${\mathrm{Cl}}_{2}$ VCD spectra to mimic spectra of the Me(II)‐(+)‐sparteine‐${\mathrm{Cl}}_{2}$ enantiomer. We then repeat the energy optimization procedure with the same input data (APT, AAT, transition dipole moments, NACs) as used in the Me(II)‐(‐)‐sparteine‐${\mathrm{Cl}}_{2}$ calculation.

**Figure 10:**
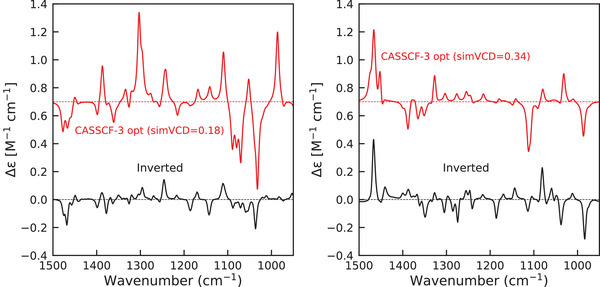
Left panel: Fitting the enhanced VCD spectra of Co(II)‐(‐)‐(sp)${\mathrm{Cl}}_{2}$ (left) to it's enantiomer. The calculated spectrum is obtained using SA‐CASSCF states and optimized excitation energies (red); the enantiomeric VCD spectrum is obtain by inversion of the original spectrum (black). Right panel: Fitting the enhanced VCD spectra of Ni(II)‐(‐)‐(sp)${\mathrm{Cl}}_{2}$ (right) to it's enantiomer.

For Co(II)(sp)${\mathrm{Cl}}_{2}$, the results of such an optimization are shown in the left panel of Figure 10. The first and foremost conclusion that can be drawn is that such an optimization leads to a simVCD value of 0.18, which is (i) clearly much lower than obtained for the correct configuration (0.70) and (ii) too low to come to a reliable assignment of the absolute configuration. Looking into more detail, we find that the optimization leads to a very low excitation energy of 0.08 eV for the first excited state. With this energy, the intensities of the resulting calculated spectrum are dramatically overestimated, especially in the 1100–1000 ${\mathrm{cm}}^{-1}$ region. Both aspects indicate that it is not possible to fit the observed experimental spectrum with the calculated input of the opposite enantiomer.

For Ni(II)(sp)${\mathrm{Cl}}_{2}$, (Figure 10, right) energy optimization similarly leads to a smaller simVCD value though less pronounced than observed for the Co complex (0.34 versus 0.51). However, the discrepancies in the overall shape of the fitted spectrum are very significant in 1400–1100 ${\mathrm{cm}}^{-1}$ region. Further support for the conclusion that the observed spectrum cannot be attributed to the enantiomer—as one would conclude if one would not know the absolute configuration of the complex—is the observation that optimization reduces the excitation energy of the third excited state to a low value of 0.18 eV while the first two states do not contribute (see Figure S6). Such an inversion in state ordering would indicate unlikely large errors in SA‐CASSCF predictions.

We thus conclude that fitting the spectrum of the opposite enantiomer with the proposed optimization procedure leads to results that can readily be identified as being inconsistent with the experiment. This makes it possible to rely on the results of the tuned enhanced calculation to come to a robust and unambiguous assignment of the absolute configuration, provided that the set of low‐lying states used in the tuning is carefully selected and validated.

### Understanding the Enhancement Mechanism

We conclude our analysis by pointing out that another advantage of the formalism presented in this work is that it offers the possibility to come to a more detailed understanding of the enhancement mechanism. To this end, we notice that we can distinguish enhancement due to the magnetic and electric dipole components of the electronic transition moments:

$$R_{i}^{enh}=Im\left[E_{i}^{tot}\cdot M_{i}^{enh}+E_{i}^{enh}\cdot M_{i}^{tot}\right].$$

**Figure 11:**
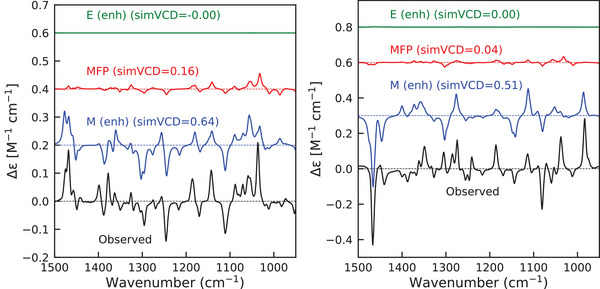
Decomposition of enhanced VCD spectra into MFP (red), electric dipole enhancement (green) and magnetic dipole enhancement (blue) for Co(II)(sp)${\mathrm{Cl}}_{2}$ (left) and Ni(II)(sp)${\mathrm{Cl}}_{2}$ (right) calculated with SA‐CASSCF excited states properties and optimized geometries.

For the case at hand, we find for all the calculated enhanced spectra that the electric dipole contribution is negligible. This is as expected since electric dipole transition moments associated with pure d–d transitions are formally forbidden (Laporte selection rule). The decomposition of the enhanced spectra into contributions for Co(II)(sp)${\mathrm{Cl}}_{2}$ and Ni(II)(sp)${\mathrm{Cl}}_{2}$ is demonstrated in Figure 11. We clearly observe the magnetic dipole enhancement dominating the signal, making the accuracy of the APT tensor (from the MFP calculation) and the magnetic dipole transition moments between electronic states more critical than the AAT tensor and the electric dipole transition moments. Although for the present case such a result is as expected, for a more general case such a decomposition will clearly contribute to a further elucidation of the factors that contribute to an observed enhancement.

## Conclusion

In this work, we have applied the sum‐over‐states approach introduced by Nafie^[^ ^32^ ^]^ to calculate enhancements of band in VCD spectra of transition metal complexes. We find that these enhancements are extremely sensitive to the excitation energies, making it challenging for quantum chemistry methods to predict these values with sufficient accuracy. Rather than pushing for even more accurate excited state calculations, we propose treating these key excitation energies as adjustable parameters. By optimizing just a few values, we can reproduce experimental VCD spectra with a high similarity, achieving simVCD values over 0.4 for both SA‐CASSCF and TDDFT.

At the CASSCF level, only two excited states for the cobalt complex and one for the nickel complex are needed to reproduce the main features of the enhanced VCD spectra, demonstrating that the sum‐over‐states expansion converges rapidly in these systems. Including different excited states can change the sign of a given vibrational transition, which highlights the importance of selecting the appropriate states in the enhancement calculation.

Our results indicate that TDDFT can perform comparably to CASSCF, provided that an appropriate functional is chosen. In our study, BHandH delivers the best VCD enhancements, likely due to its high fraction of Hartree‐Fock exchange, which is crucial for accurately describing d–d transitions in transition metal complexes. In fact, for Ni(II)(sp)${\mathrm{Cl}}_{2}$, the non‐adiabatic couplings calculated with BHandH closely match those of SA‐CASSCF, leading to nearly identical spectra with SA‐CASSCF states. For Co(II)(sp)${\mathrm{Cl}}_{2}$, we observe some differences in the couplings to the first and third excited states between the two methods, accounting for the slight differences in their respective contributions.

An important observation noted in earlier studies as well is the local character of the VCD enhancement. Here, we show that significant non‐adiabatic couplings occur only between the transition metal and its close neighbors: two nitrogens and two chlorines in the sparteine case. Consequently, we assume that for larger systems, one could compute these couplings on a truncated model without substantial loss of accuracy. We plan to explore this idea in future work. A further direction we are working on concerns the fact that in the present study we have studied rigid sparteine complexes as a proof‐of‐concept application of vibronic coupling theory. The next step will be to extend this approach to flexible complexes, where conformational variability introduces additional complications.

## Conflict of Interests

The authors declare no conflict of interest.

## Author Contributions

**Mariia Sapova**: Conceptualization; Data Curation; Formal Analysis; Investigation; Methodology; Software; Writing — original draft; Writing — review & editing. **Chandan Kumar**: Data Curation; Investigation; Writing — original draft. **Sahar Ashtari‐Jafari**: Data Curation; Investigation. **Wybren J. Buma**: Conceptualization; Funding acquisition; Methodology; Project Administration; Resources; Supervision; Writing — review & editing. **Lucas Visscher**: Conceptualization; Funding acquisition; Methodology; Project Administration; Resources; Supervision; Writing — original draft; Writing — review & editing.

## Supporting information

Supporting Information

## Data Availability

The data that support the findings of this study are available in the supplementary material of this article.

## Footnotes

1 In passing by, we note that a similar assumption is made as well in the MFP formalism but in that case its repercussions can be expected to be much less.
