# Supplementary material for: Absolute Configuration Determination with Electronically Enhanced Vibrational Circular Dichroism
Source: Angew Chem Int Ed Engl. 2025 Nov 17;65(1):e17979. doi: 10.1002/anie.202517979 (PMC12759258; doi:10.1002/anie.202517979)
Supplement: Supplementary file 1 — Supporting Information [file ANIE-65-e17979-s001.pdf]

Supporting Information: Absolute Configuration Determination with Electronically Enhanced Vibrational Circular Dichroism

Mariia Sapova,[a] Chandan Kumar,[b] Sahar Ashtari-Jafari,[a] Wybren J. Buma,*[b, c] Lucas Visscher,*[a]

Contents

S1. Computational Details 1

S2. Magnetic Field Perturbation Calculations 2

S3. Excited State Calculations 6
S3.1. CASSCF Orbital Composition 7
S3.2. GGA Functionals 9
S3.3. Global Hybrid and Range-Separated Functionals 10

S4. Enhanced VCD Calculations 11
S4.1. Intensity Normalization 11
S4.2. Enantiomer Contributions 11
S4.3. SA-CASSCF 12
S4.4. B3LYP Functional 13
S4.5. BP86 Functional 14

S5. Non-Adiabatic Couplings 15
S5.1. $Co(sp)Cl_2$ 15
S5.2. $Ni(sp)Cl_2$ 16
S5.3. NAC Analysis 17

S6. Coordinates 18

S1. Computational Details

According to previous studies [1], the sparteine complexes are inherently rigid as a result of the ligand structure itself. To provide an independent verification of this finding, we use CREST [2] within the Amsterdam Modeling Suite (AMS) [3] to generate initial conformers of $Zn(sp)Cl_2$ employing the GFN1-xTB method [4]. Conformers within 5 kcal/mol of the lowest energy structure are subsequently reoptimized at the DFT level using BP86 [5,6]-D3BJ [7,8]/def2-TZVP with the conductor-like polarizable continuum model (CPCM) [9] to model $CHCl_3$ solvent effects. All the IR and MFP VCD calculations are performed on the same level of theory for closed-shell $Zn(sp)Cl_2$ and open-shell $Co(sp)Cl_2$ and $Ni(sp)Cl_2$ complexes.

We calculate excited states of open-shell complexes using CASSCF and TDDFT, including dipole transition moments (EDTM/MDTM) and non-adiabatic couplings. TDDFT calculations are performed with ORCA 6.0 [10]. We use a def2-TZVP [11] basis set with def2/JK [12] auxiliary sets and extract electric and magnetic transition dipole moments (EDTM/MDTM) and non-adiabatic couplings for further enhancement calculations. CASSCF calculations are performed with both ORCA 6.0 and Openmolcas v23.06 [13]. In ORCA, we perform state-averaged (SA-) and state-specific (SS-) CASSCF on sparteine and model C_{2v} complexes with the def2-TZVP basis set and the def2/JK auxiliary fitting. OpenMolcas is employed specifically for its implementation of non-adiabatic coupling vectors at the SA-CASSCF level. There we use the ANO-RCC-VTZP basis on the metal [14], N, and Cl atoms [15], and the minimal ANO-RCC-MB basis on C and H.

We calculate enhanced VCD spectra using our developed Python implementation of Nafie's vibronic coupling theory. To quantify the agreement of the calculated IR and VCD spectra with the observed ones we use Shen [16] simIR and simVCD metrics. To this end, we digitized the experimental IR and VCD spectra for all sparteine complexes from the thesis of He [17] using a Python script. A frequency scaling factor is obtained by maximizing the simIR between the computed and experimental IR spectra over the 950-1500 cm^{-1} window. The same factor is then applied to the calculated VCD spectra.

S2. Magnetic Field Perturbation Calculations

Zn(II) has a closed-shell d^{10} configuration with high-lying excited states. Enhancement effects are in this case thus negligible and the MFP approximation is fully justified. As a result, this complex serves as a good benchmark for MFPbased VCD calculations. The IR spectra of all three studied Me(II)(sp)Cl_2 (M = Zn, Co, Ni) are almost identical, so the Zn(sp)Cl_2 complex can be used to select the most appropriate DFT functional and to evaluate conformational effects. Conformational analysis reveals an energy difference between the two lowest energy conformers of Zn(sp)Cl_2 of 4.2 kcal/mol which exceeds the value of 3 kcal/mol typically used to select contributing conformers. This is in agreement with previous studies of the sparteine ligand [1] where it was also found that Me(II)(sp)Cl_2 complexes exist predominantly in a single conformation, which makes the systems ideal for analysing their VCD spectra in detail.

Several benchmark studies have shown that the generalized gradient approximation (GGA) functional BP86 [5,18] outperforms B3LYP [19] in reproducing experimental equilibrium geometries of small first-row transition metal complexes [20,21]. Minenkov et al. [22] furthermore reported that B3LYP systematically overestimates the metal-ligand bonds in transition metal complexes of the first and second row. A more recent benchmark for metal-dinitrogen complexes [23] further showed that the performance of DFT is highly sensitive to the nature of the N_2 coordination mode at the metal center.

In view of these observations and to establish the optimal computational approach for the present system we compare in the following the performance of three DFT functionals (BP86, B3LYP, PBE0) [24] with and without Grimmes D3(BJ) dispersion correction [7,8] in the gas phase and with CPCM of $CHCl_3$ [9]. Figure S1 and Figure S2 show calculated IR and MFP-VCD spectra of Zn(sp)Cl_2 with the considered functionals. The addition of the dispersion correction improves both simIR and simVCD for BP86 and B3LYP (see Figure S1). BP86-D3BJ/def2-TZVP with CPCM demonstrates the best agreement with the experiment with good similarities for both IR and VCD (simIR = 0.88, simVCD = 0.44), noticing that simVCD values larger than 0.4 are typically considered to be secure to assign the absolute configuration [25]. Figure S3 shows that this level of theory predicts IR spectra for the open-shell Co(sp)Cl_2 and Ni(sp)Cl_2 systems that also have a high similarity to the experiment. We thus conclude that calculations at the BP86-D3BJ/def2-TZVP with CPCM level of theory are suitable to optimize the ground state structure of these compounds and compute their IR spectra.

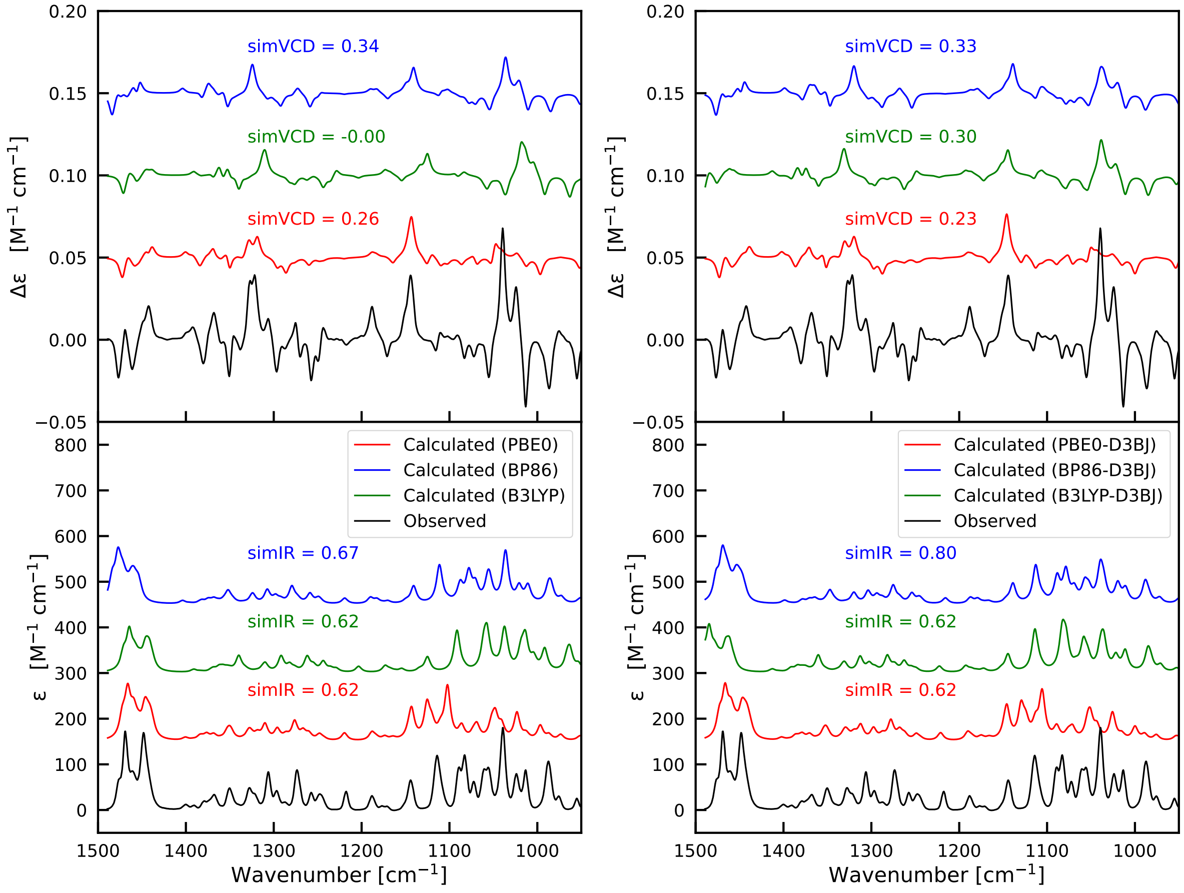

Figure S1. Comparison of VCD (top) and IR (bottom) spectra of Zn(sp)Cl_2. Experimental spectra are given in black. All VCD spectra are calculated using frequency scaling factors from maximizing simIR values. For the B3LYP [19] functional simVCD is approximately zero, however, using a separate frequency scaling factor for VCD would give simVCD = 0.22. Calculation are performed with Orca [10] using the def2-TZVP [11] basis sets with def2/JK [12].

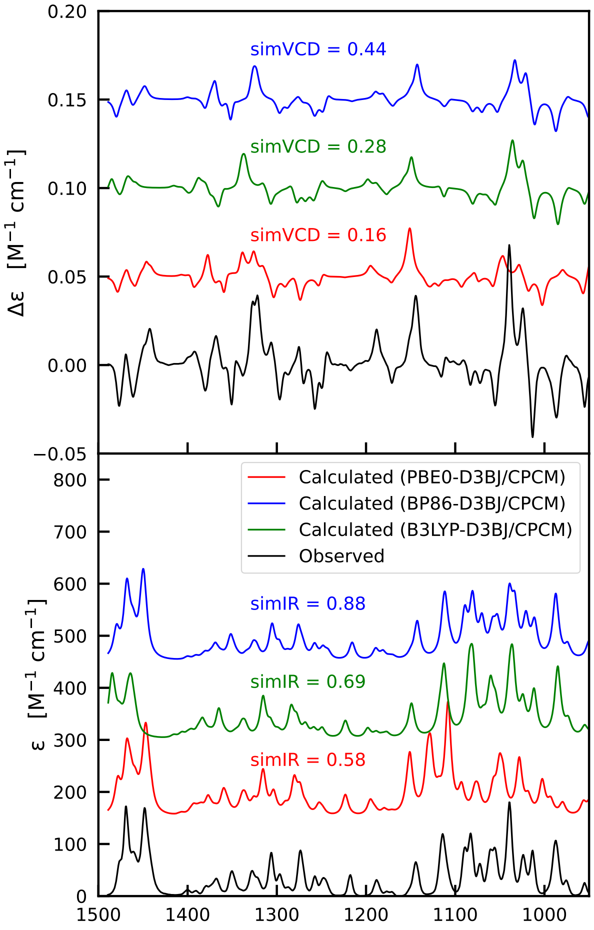

Figure S2. Comparison of VCD (top) and IR (bottom) spectra of Zn(sp)Cl_2. Experimental spectra are given in black, the spectra are calculated with CPCM of $CHCl_3$ with PBE0/def2-TZVP (red, simVCD = 0.16), B3LYP/def2-TZVP (green, simVCD = 0.28), and BP86/def2-TZVP (blue, simVCD = 0.44).

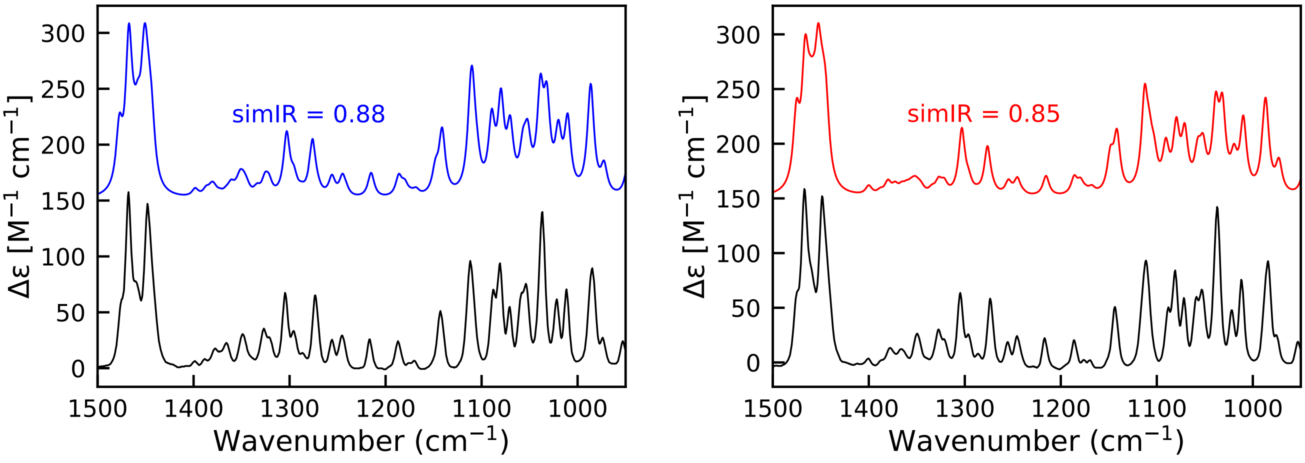

Figure S3. Left panel: calculated (blue, simIR = 0.88) and observed (black) IR spectra of Co(II)(sp)Cl_2. Right panel: calculated (red, simIR = 0.85) and observed (black) IR spectra of Ni(II)(sp)Cl_2. All calculations have been performed with BP86-D3BJ/def2-TZVP with CPCM of $CHCl_3$.

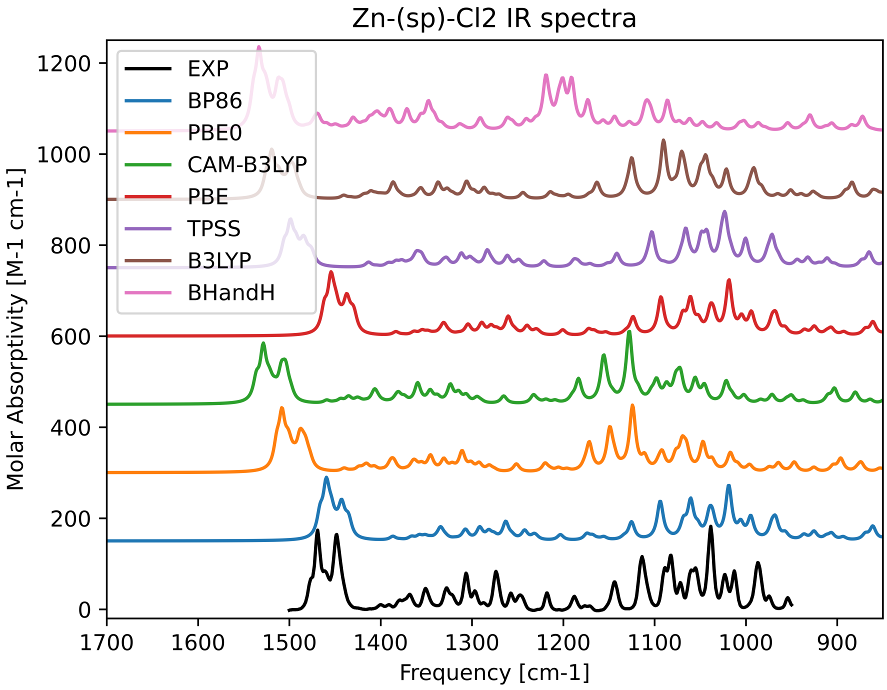

Figure S4. IR spectra calculated for Zn(sp)Cl_2 with various DFAs. All the calculations are performed with ADF [26] (version 2023) on DFA/TZP preoptimized geometries. No frequency scaling factor is applied.

S3. Excited State Calculations

Table S1. Comparison of excitation energies for Co(II) and Ni(II) sparteine complexes with various DFT functionals calculated with ORCA [10] on the BP86-D3(BJ)-CPCM($CHCl_3$) [7,8] optimized geometry. All energy values are given in eV.

	**Co(II)(sp)Cl$_2$**					**Ni(II)(sp)Cl$_2$**				
#.	BP86	B3LYP	PBE0	BHandH	M06	BP86	B3LYP	PBE0	BHandH	M06
1	0.92	0.83	0.82	0.47	0.13	0.79	0.77	0.77	0.43	0.06
2	0.95	0.86	0.85	0.51	0.27	1.10	1.04	1.02	0.66	0.54
3	1.08	1.12	1.11	0.77	0.39	1.47	1.46	1.42	1.00	0.91
4	1.41	1.49	1.51	1.38	0.57	1.66	1.70	1.76	1.62	1.23
5	1.63	1.63	1.65	1.50	0.79	1.72	1.87	1.91	1.75	1.40
6	2.00	2.12	2.13	1.98	1.11	1.84	2.03	2.07	1.90	1.47

Table S2. Comparison of excitation energies for Co(II)(sp)Cl$_2$ calculated with Orca on SA-CASSCF/NEVPT2 level with different active space size. All energy values are given in eV.

#.	CAS(7, 5)/NEVPT2	CAS(7, 10)/NEVPT2	CAS(11, 12)/NEVPT2
1	0.33 (0.42)	0.34 (0.39)	0.35 (0.39)
2	0.36 (0.47)	0.37 (0.43)	0.42 (0.46)
3	0.41 (0.52)	0.42 (0.47)	0.49 (0.50)
4	0.67 (0.86)	0.70 (0.80)	0.77 (0.83)
6	0.84 (1.05)	0.87 (0.97)	0.96 (1.02)
7	1.08 (1.36)	1.13 (1.27)	1.24 (1.34)

Table S3. Comparison of excitation energies for Ni(II)(sp)Cl$_2$ calculated with Orca on SA-CASSCF/NEVPT2 level with different active space size. All energy values are given in eV.

#.	CAS(8, 5)/NEVPT2	CAS(12, 12)/NEVPT2
1	0.30 (0.39)	0.42 (0.43)
2	0.47 (0.61)	0.60 (0.63)
3	0.70 (0.92)	0.85 (0.92)

Table S4. Comparison of adiabatic (adiab) and vertical (vert) excitation energies for Co(II)(sp)Cl$_2$ and Ni(II)(sp)Cl$_2$ calculated with SA-CASSCF and NEVPT2 corrections (given in parenthesis), minimal CAS. Adiabatic energies are calculated with SA-CASSCF/NEVPT2 on optimized ground and excited state geometries from BP86-D3BJ (BP86 in table) and BHandH functionals. All energy values are given in eV.

	BP86	adiab	vert	BHandH	adiab	vert
Co(II)(sp)Cl$_2$	1	0.16 (0.25)	0.34 (0.42)	1	0.16 (0.26)	0.32 (0.40)
	2	0.43 (0.56)	0.37 (0.48)	2	0.26 (0.44)	0.37 (0.48)
	3	0.29 (0.39)	0.42 (0.53)	3	0.19 (0.38)	0.41 (0.51)
	BP86	adiab	vert	BHandH	adiab	vert
Ni(II)(sp)Cl$_2$	1	0.30 (0.38)	0.30 (0.39)	1	0.12 (0.24)	0.31 (0.39)
	2	0.77 (1.04)	0.47 (0.62)	2	0.32 (0.62)	0.49 (0.65)
	3	0.55 (0.86)	0.70 (0.92)	3	0.40 (0.81)	0.75 (0.99)

S3.1. CASSCF Orbital Composition

We show a Löwdin orbital decomposition for the minimal (5 orbitals) and largest (12 orbitals) active spaces in SA-CASSCF calculations to confirm the dominant d character of low-lying excited states.

Table S5. Co(II)(sp)Cl2 SA-CASSCF(7, 5) occupation numbers, Löwdin orbital compositions and total d-character percentage.

Orbital	1	2	3	4	5
occupation	1.71	1.47	1.45	1.31	1.05
Composition	%	%	%	%	%
Co d_{z^2}	**72.0**	0.0	0.1	22.0	3.3
Co d_{xz}	3.8	0.3	10.0	0.0	**80.9**
Co d_{yz}	0.9	0.7	**83.8**	0.1	9.2
Co $d_{x^2-y^2}$	16.8	23.9	0.2	**54.4**	0.7
Co d_{xy}	4.4	**73.1**	0.9	18.9	0.5
Co d total %	97.9	98.0	95.0	95.4	94.6

Table S6. Co(II)(sp)Cl2 SA-CASSCF(11, 12) occupation numbers, Löwdin orbital compositions and total d-character percentage.

Orbital	1	2	3	4	5	6	7	8	9	10	11	12
occupation	1.999	1.999	1.43	1.42	1.39	1.38	1.35	0.008	0.008	0.008	0.008	0.007
	%	%	%	%	%	%	%	%	%	%	%	%
Co p_z	1.0	0.0	0.0	0.7	0.3	0.0	0.0	0.1	0.8	0.1	1.2	0.0
Co p_x	0.2	2.5	0.0	0.0	0.0	0.1	1.1	0.0	0.0	0.0	0.0	3.0
Co p_y	4.2	1.2	0.0	0.0	0.0	1.2	0.3	0.0	0.2	2.2	0.3	0.0
Co d_{z^2}	1.0	0.6	4.0	10.0	**77.3**	2.0	3.8	13.2	19.6	22.9	33.1	3.3
Co d_{xz}	3.0	5.6	0.0	2.8	0.0	**46.6**	**43.5**	0.1	0.3	14.8	1.0	74.0
Co d_{yz}	2.4	5.3	0.4	0.0	7.5	**41.5**	**43.2**	0.0	3.7	42.8	29.1	11.7
Co $d_{x^2-y^2}$	3.2	0.0	18.0	**62.3**	11.7	3.1	0.0	37.4	20.9	7.8	24.2	0.0
Co d_{xy}	0.5	0.0	**75.5**	20.8	0.0	0.0	1.1	42.5	47.0	0.2	3.1	0.4
Cl p_z	10.9	0.8	0.0	0.3	0.8	0.1	0.0	0.0	0.0	0.1	0.8	0.0
Cl p_x	0.0	3.8	0.5	0.0	0.0	0.0	0.5	0.4	0.2	0.0	0.0	0.4
Cl p_y	35.2	8.1	0.0	0.4	0.0	1.4	0.4	0.0	0.1	0.2	0.1	0.0
Cl p_z	0.8	1.9	0.0	0.3	0.8	0.0	0.1	0.0	0.0	0.3	0.5	0.0
Cl p_x	0.4	5.2	0.5	0.0	0.0	0.1	0.5	0.3	0.1	0.0	0.0	0.5
Cl p_y	9.7	10.0	0.0	0.1	0.0	1.0	0.6	0.0	0.0	0.4	0.1	0.0
N s	0.0	1.7	0.0	0.0	0.0	0.0	0.2	0.0	0.0	0.0	0.0	0.2
N p_z	0.1	11.6	0.0	0.2	0.1	0.1	0.7	0.1	0.1	0.0	0.1	0.2
N p_x	0.1	7.1	0.0	0.0	0.1	0.0	0.4	0.0	0.1	0.0	0.0	0.1
N p_y	0.2	0.4	0.0	0.0	0.0	0.1	0.0	0.1	0.1	0.1	0.1	0.0
N s	1.4	1.5	0.0	0.0	0.0	0.1	0.2	0.0	0.0	0.0	0.0	0.2
N p_z	6.3	6.2	0.0	0.2	0.0	0.2	0.4	0.1	0.1	0.1	0.0	0.1
N p_x	9.6	11.2	0.0	0.1	0.1	0.3	0.8	0.0	0.1	0.0	0.0	0.2
N p_y	1.7	0.5	0.0	0.0	0.0	0.1	0.0	0.1	0.0	0.2	0.0	0.0
Co d total %	10.1	11.5	97.9	95.9	96.5	93.2	91.6	93.2	91.5	88.5	90.5	89.4

Table S7. Ni(II)(sp)Cl2 SA-CASSCF(8, 5) occupation numbers, Löwdin orbital compositions and total d-character percentage.

orbital	1	2	3	4	5
occupation	1.82	1.74	1.67	1.45	1.32
	%	%	%	%	%
Ni d_{z^2}	**51.9**	13.9	8.6	11.4	12
Ni d_{xz}	0.1	0.1	17.0	16.7	**61.2**
Ni d_{yz}	15.2	9.1	**53.6**	0.8	17.1
Ni $d_{x^2-y^2}$	0.8	16.2	17.0	**58.0**	3.8
Ni d_{xy}	30.2	**59.0**	0.3	8.0	0.5
Ni d total %	98.2	98.3	96.5	94.9	94.6

Table S8. Ni(II)(sp)Cl2 SA-CASSCF(12, 12) occupation numbers, Löwdin orbital compositions and total d-character percentage.

orbital	1	2	3	4	5	6	7	8	9	10	11	12
occupation	1.999	1.999	1.792	1.744	1.675	1.422	1.324	0.010	0.010	0.010	0.008	0.007
	%	%	%	%	%	%	%	%	%	%	%	%
Ni s	0.7	0	0	0	0.1	0.1	0	0	0.2	0.2	0.2	0
Ni p_z	3.8	0	0	0	0.1	1	0.1	0	0	0.2	1.9	0.2
Ni p_x	0	3	0	0	0	0.2	1.1	0	0	0	0.3	2.3
Ni p_y	0.8	0.1	0.1	0	0.5	0.2	0	0.2	0	1.1	0.3	0
Ni d_{z^2}	2.8	0.7	**42.1**	19.1	11.8	13.4	10.6	39.3	21.8	6.6	16.9	9.3
Ni d_{xz}	0.2	9	0	0	16.7	11.8	**64.1**	0	0.2	14.5	10	67.5
Ni d_{yz}	0.1	2.4	16.1	10.9	**54.1**	0.3	13.8	13.3	5.4	59.1	0.3	12.4
Ni $d_{x^2-y^2}$	7.9	0	0.1	21.3	13	**57.0**	2.4	0.3	26.6	8.3	54.3	1.7
Ni d_{xy}	1.1	0	**39.9**	**47.0**	0.9	9.5	0.5	41.3	40.8	2.6	9.4	0.3
Cl p_z	14.1	0.3	0.1	0	0.1	0.9	0.1	0.1	0.1	0.2	0.3	0
Cl p_x	0.1	2.4	0.1	0.3	0	0	0.3	0.2	0.3	0	0.1	0.2
Cl p_y	25.4	1.9	0.1	0.1	0.2	1.2	0.3	0.1	0	0.1	0.2	0
Cl p_z	16.2	0	0.1	0	0.1	1.2	0.1	0	0	0.1	0.5	0.1
Cl p_x	0	4	0.2	0.2	0	0	0.5	0.2	0.2	0	0	0.4
Cl p_y	0.7	2.8	0	0	0.8	0	0.2	0	0	0.3	0.1	0
N s	0.4	2.3	0	0	0	0	0.4	0	0	0	0	0.2
N p_z	3.4	15.8	0	0	0	0.1	1.3	0.1	0.1	0	0	0.2
N p_x	1.5	9.8	0	0	0	0	0.8	0	0	0.1	0	0.1
N p_y	0.2	1	0	0	0.1	0	0.1	0.2	0	0.2	0	0
N s	1	1.9	0	0	0	0.2	0.2	0	0	0	0.1	0.1
N p_z	4.9	7.5	0	0	0	0.5	0.3	0.1	0	0.1	0.1	0.1
N p_x	7	14.4	0	0	0	0.8	0.8	0	0	0	0.1	0.2
N p_y	0.8	1	0	0	0	0.1	0	0	0.1	0.1	0	0
Ni d total %	12.1	12.1	98.2	98.3	96.5	92.0	91.4	94.2	94.8	91.1	90.9	91.2

S3.2. GGA Functionals

Table S9. Excitation energies of Co((II)sp)Cl_2 complex in eV calculated with GGA DFAs (see DFA references on the SCM website). The calculations were performed with ADF[26] (2019 version) for the lowest 10 electronic transitions on the BP86/TZP preoptimized geometry using TZP basis sets.

#.	BP86	PBE	mPBE	revPBE	OPBE	PW91
1	1.086	1.048	1.079	1.133	1.033	1.069
2	1.108	1.066	1.097	1.148	1.043	1.090
3	1.187	1.146	1.176	1.230	1.127	1.169
4	1.626	1.584	1.614	1.667	1.548	1.608
5	1.778	1.729	1.758	1.811	1.681	1.755
6	1.998	1.952	1.982	2.036	1.910	1.975
7	2.864	2.879	2.896	2.926	2.995	2.856
8	3.002	3.017	3.035	3.067	3.128	2.994
9	3.148	3.161	3.177	3.208	3.260	3.140
10	3.179	3.185	3.205	3.240	3.268	3.168

#.	mPw	BLYP	OLYP	LB94	KT1	KT2
1	1.118	1.074	1.022	0.384	1.496	1.644
2	1.140	1.100	1.028	0.463	1.541	1.685
3	1.219	1.171	1.106	0.502	1.631	1.772
4	1.658	1.611	1.529	0.885	2.083	2.225
5	1.807	1.753	1.654	0.972	2.257	2.390
6	2.027	1.975	1.885	1.142	2.460	2.594
7	2.869	2.805	2.915	1.885	3.079	3.139
8	3.009	2.948	2.927	1.964	3.228	3.296
9	3.154	3.086	3.054	2.151	3.386	3.441
10	3.188	3.123	3.073	2.174	3.443	3.522

S3.3. Global Hybrid and Range-Separated Functionals

Table S10. Excitation energies of Co(sp)Cl_2 complex in eV calculated with hybrid and range-separated DFAs (see DFA references on the SCM website). The calculations were performed with ADF[26] (2019 version) for the lowest 10 electronic transitions on the BP86/TZP optimized geometry using TZP basis sets.

#.	PBE0	OPBE0	B3LYP	O3LYP	X3LYP	BHandH
1	0.937	0.895	0.959	0.485	0.939	0.258
2	1.015	0.978	1.030	0.553	1.011	0.334
3	1.141	1.107	1.134	0.605	1.125	0.514
4	1.593	1.540	1.576	1.050	1.567	1.144
5	1.719	1.690	1.688	1.156	1.682	1.301
6	2.103	2.057	2.060	1.467	2.061	1.707
7	4.486	4.647	3.967	3.230	4.090	6.155
8	4.707	4.864	4.188	3.415	4.312	6.236
9	4.717	4.881	4.197	3.479	4.319	6.358
10	4.765	4.915	4.259	3.494	4.380	6.381

#.	BHandHLYP	B1PW91	mPW1PW	CAMY-B3LYP	WB97X	CAM-B3LYP
1	0.710	0.982	1.005	0.939	0.785	0.902
2	0.773	1.061	1.084	1.019	0.868	0.986
3	0.948	1.187	1.209	1.130	0.977	1.099
4	1.604	1.640	1.664	1.572	1.403	1.537
5	1.744	1.768	1.790	1.685	1.506	1.648
6	2.158	2.152	2.175	2.061	1.876	2.025
7	6.379	4.484	4.476	4.427	4.882	4.639
8	6.457	4.708	4.700	4.636	5.048	4.837
9	6.583	4.714	4.707	4.678	5.167	4.901
10	6.607	4.765	4.759	4.721	5.275	4.936

S4. Enhanced VCD Calculations

S4.1. Intensity Normalization

We show MFP and enhanced VCD spectra with normalized intensities to facilitate comparison to the observed VCD band shapes. In terms of band shapes, the largest discrepancies between the MFP spectra and the experiment occur near the electronic transitions (on the left side of the spectra), which is consistent with our expectation that vibronic coupling primarily affects this region. For the Co(II)(sp)Cl_2 complex the MFP shape is still rather close to the observed shape, but for Ni(II)(sp)Cl_2 deviations are very significant.

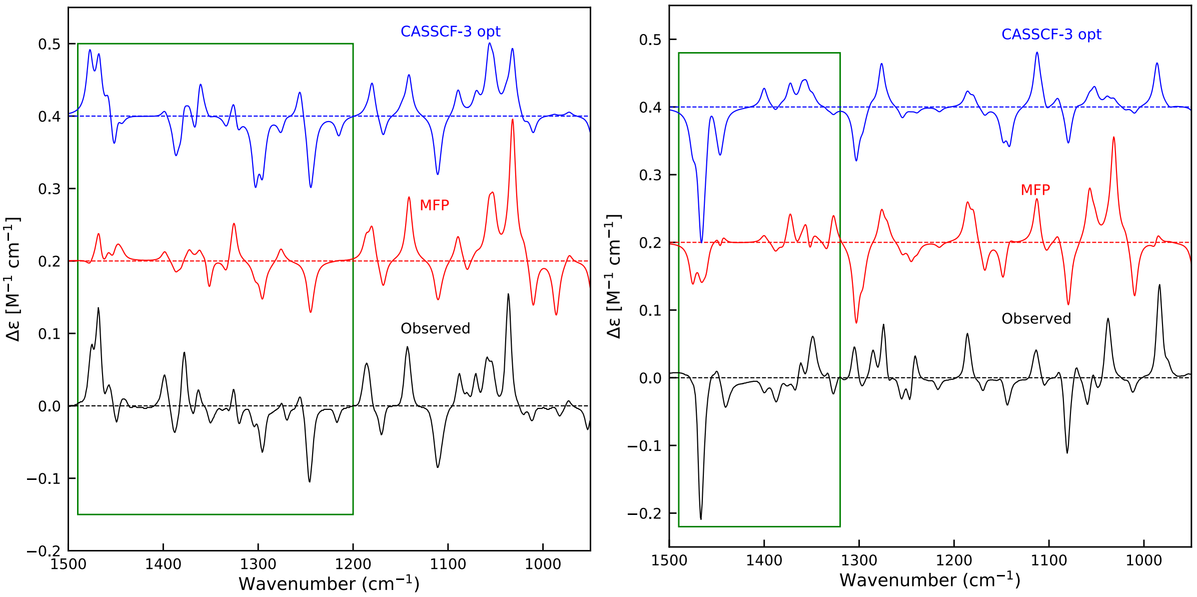

Figure S5. Normalized VCD spectra MFP (red), calculated enhanced with SA-CASSCF including three LLESs with optimized energies (blue) and observed spectra for Co(sp)Cl_2 (left) and Ni(sp)Cl_2 (right).

S4.2. Enantiomer Contributions

We here plot individual contributions of each excited state when fitting the VCD spectra of calculated Me(II)-(-)-sparteine-Cl_2 to enatiomeric Me(II)-(+)-sparteine-Cl_2. This analysis is carried out to check the reliability of the energy fitting process and provides a guarantee against overfitting. In the Co(II) case, trying to fit the spectrum of the other enantiomer leads to an improbably low excitation energy from the first excited state with a huge overestimation of the intensities. For Ni(II) case only the first peak around 1450 cm^{-1} can be well-fitted, while the overall shape of the fitted and experimental spectra remains significantly different. The first two states do not contribute in this case, which would suggest an unlikely reordering of the SA-CASSCF predicted energies. This analysis demonstrates the usefulness of sanity checks in the fitting workflow and supports the reliability of absolute configuration assignments based on our procedure.

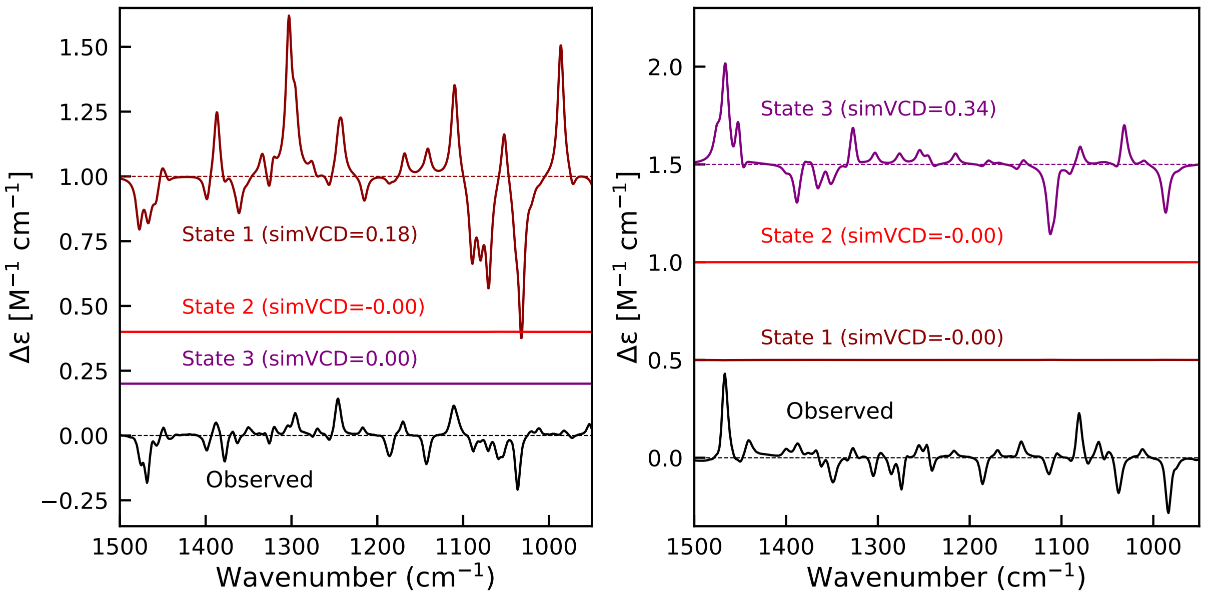

Figure S6. Individual state contributions of fitting Co(II)-(-)-(sparteine)-Cl_2 (left) and Ni(II)-(-)-(sparteine)-Cl_2 to enantiomeric Me(II)-(+)-(sparteine)-Cl_2 VCD spectra.

S4.3. SA-CASSCF

As we mention in the main text optimization of energies in enhanced VCD spectra with SA-CASSCF leads the third state to the upper bound value. This is happening because the third state has a negative similarity with observed spectra.

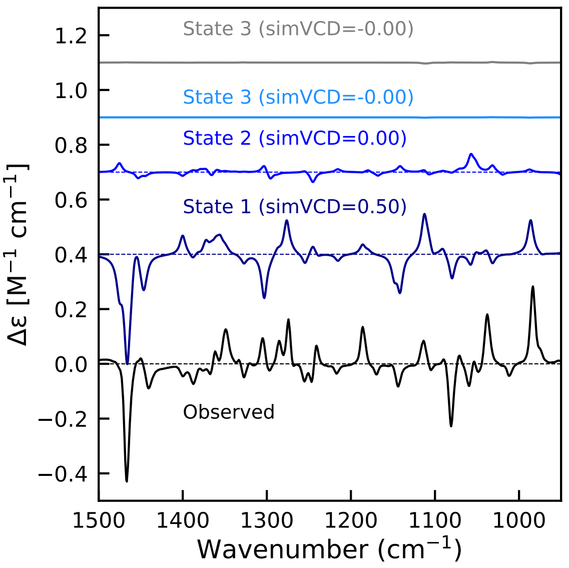

Figure S7. Enhanced VCD spectra of Ni(II)(sp)Cl_2 with SA-CASSCF decomposition into state contributions. Three states with optimized energies are shown in blue color. The grey curve shows the third state contribution if we set it to 0.70 eV (value from SA-CASSCF calculation). Since the third state does not contribute to enhancement, we can't identify it's energy from the approach.

S4.4. B3LYP Functional

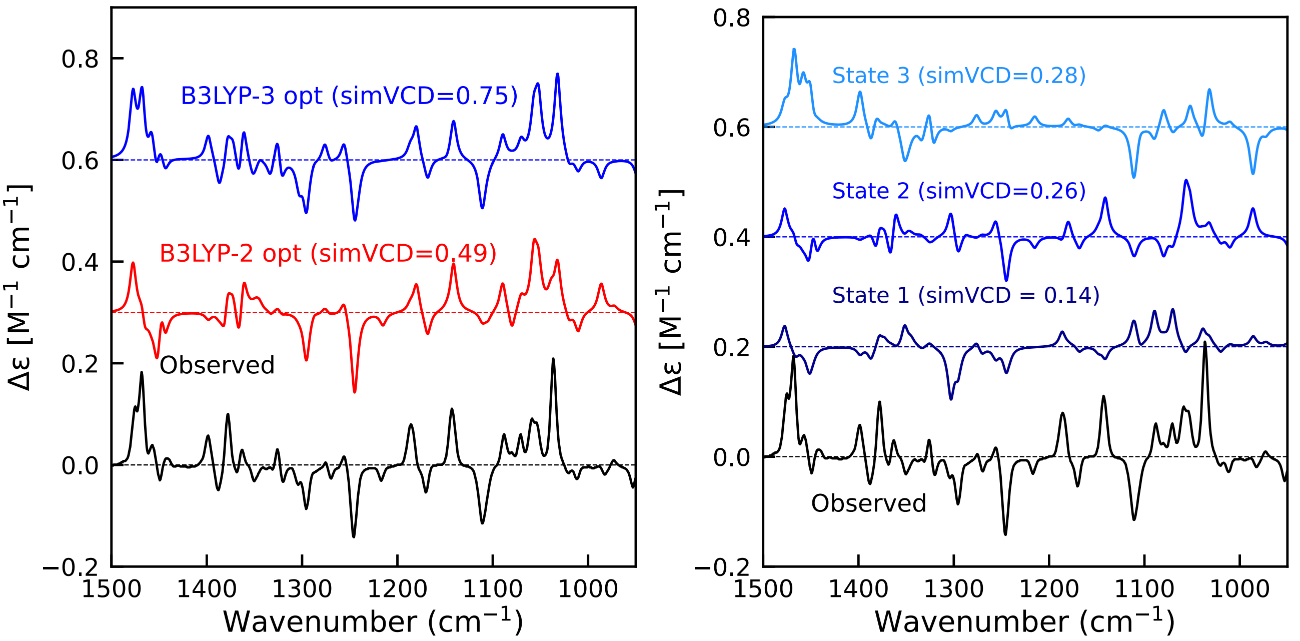

Figure S8. Enhanced VCD spectra of Co(II)(sp)Cl_2 calculated with the B3LYP functional including two (red) and three (blue) excited states. The right panel shows individual contributions of the excited states to the B3LYP-3 opt spectra. The excitation energies of the three states after optimization are all around 0.23 eV (similar to BHandH result).

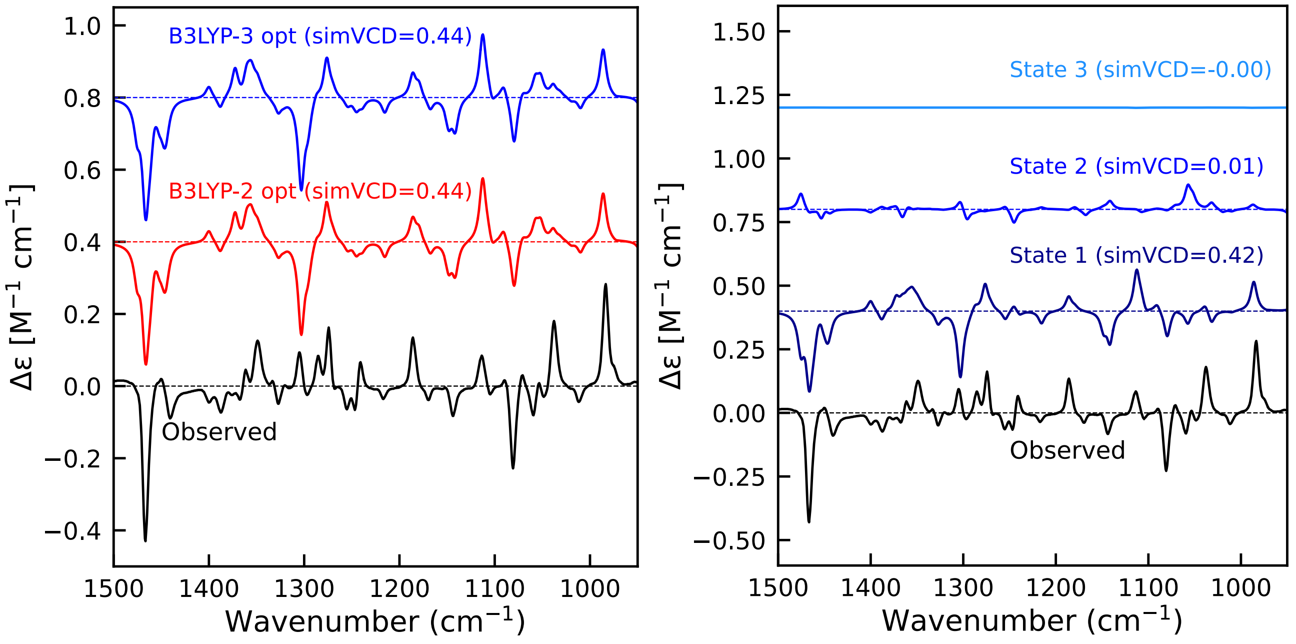

Figure S9. Enhanced VCD spectra of Ni(II)(sp)Cl_2 calculated with the B3LYP functional including two (red) and three (blue) excited states. The right panel shows individual contributions of the excited states to the B3LYP-3 opt spectra. The energy of the first excited state is 0.22 eV (similar to BHandH result).

S4.5. BP86 Functional

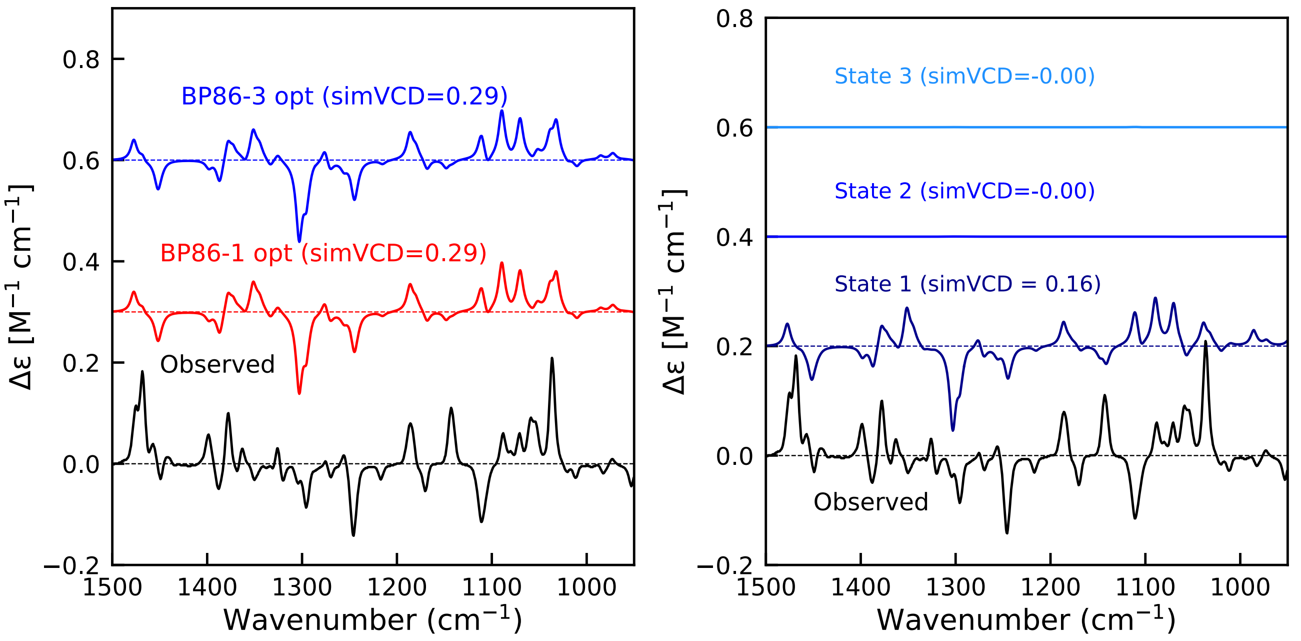

Figure S10. Enhanced VCD spectra of Co(II)(sp)Cl_2 calculated with the BP86 functional. The excitation energies of the three states after optimization are 0.20, 1.0 and 1.0 eV, where the second and the third state do not conitribute. The first state energy is close to the SA-CASSCF result, however, since there is no degeneracy with the second state, in fact only the first state is responsible for the enhancement effect. Here the 1200-1100 cm^{-1} is significantly different from the observed spectra, and the fine structure is missing in 1500-1300 cm^{-1}, which overall sums into simVCD smaller that with BHandH and B3LYP.

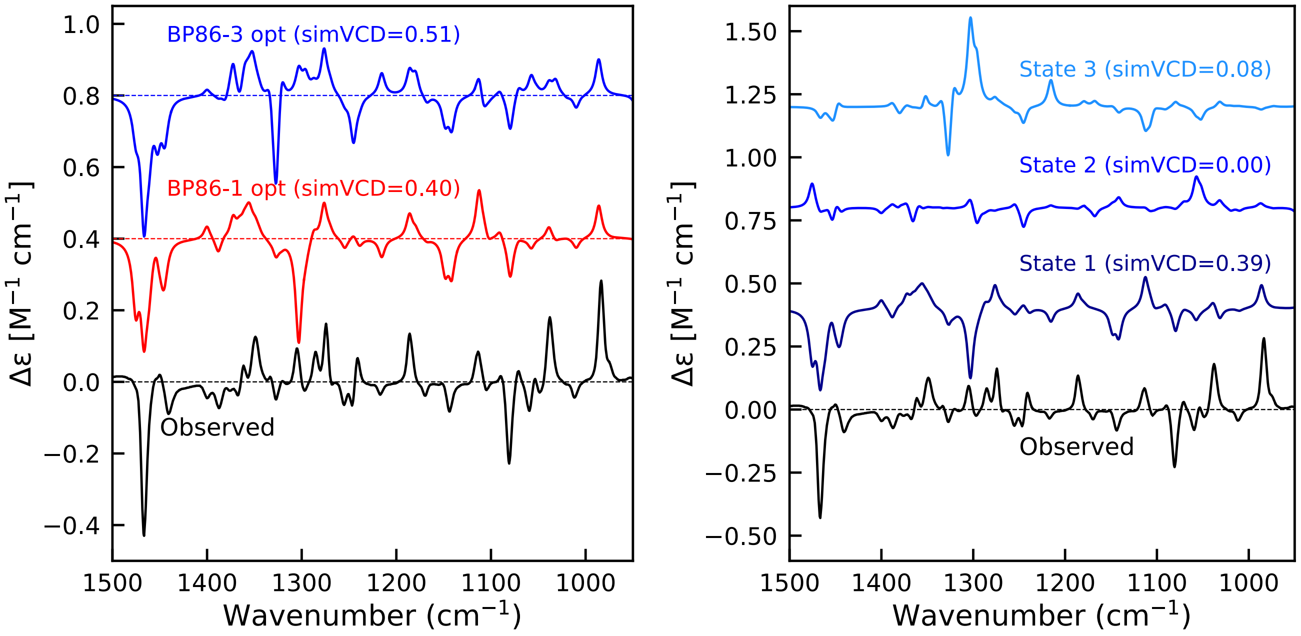

Figure S11. Enhanced VCD spectra of Ni(II)(sp)Cl_2 calculated with the BP86 functional. The excitation energies of the two states after optimization are 0.2 eV and 0.22 eV, and the third state is low in energy 0.16 eV. The significant differences in state conitrbutions comes probably from NACME differences from BP86 and SA-CASSCF/BHandH/B3LYP. The improved simVCD value for three states (BP86-3) comes from a significant positive peak in the third state at 1300 cm^{-1}, which compensates the negative peak from the first excited state.

S5. Non-Adiabatic Couplings

S5.1. Co(sp)Cl_2

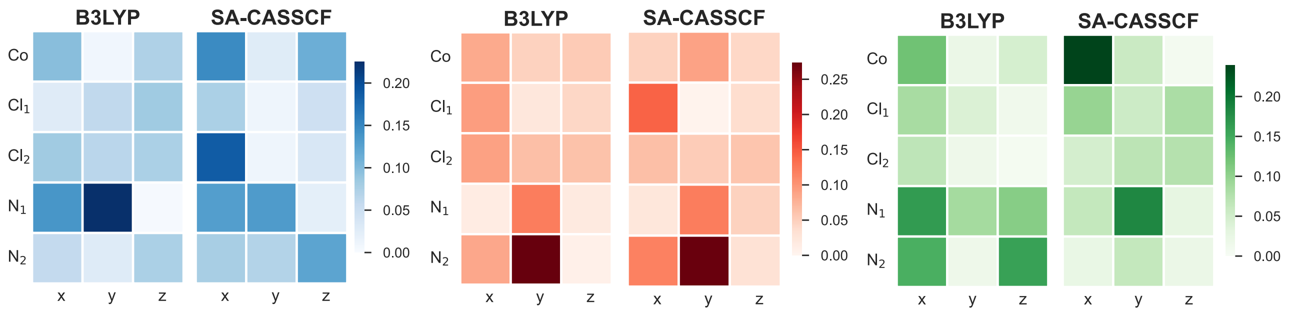

Figure S12. Non-adiabatic couplings between the ground and first three excited states of Co(II)(sp)Cl_2 calculated with B3LYP and SA-CASSCF.

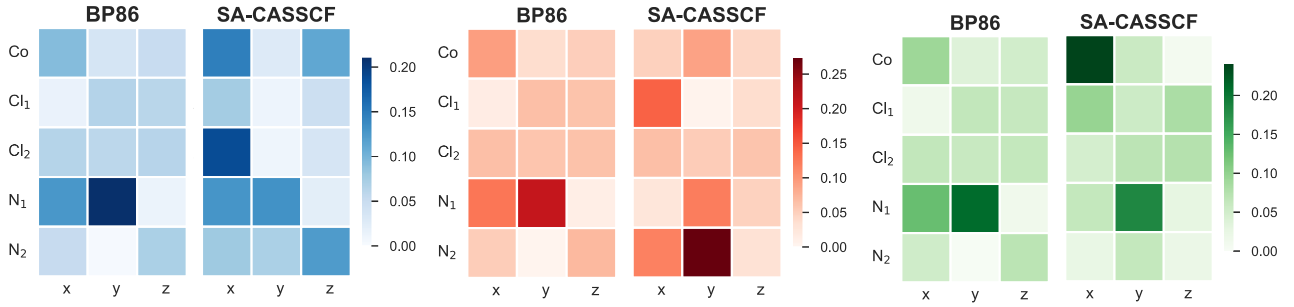

Figure S13. Non-adiabatic couplings between the ground and first three excited states of Co(II)(sp)Cl_2 calculated with BP86 and SA-CASSCF.

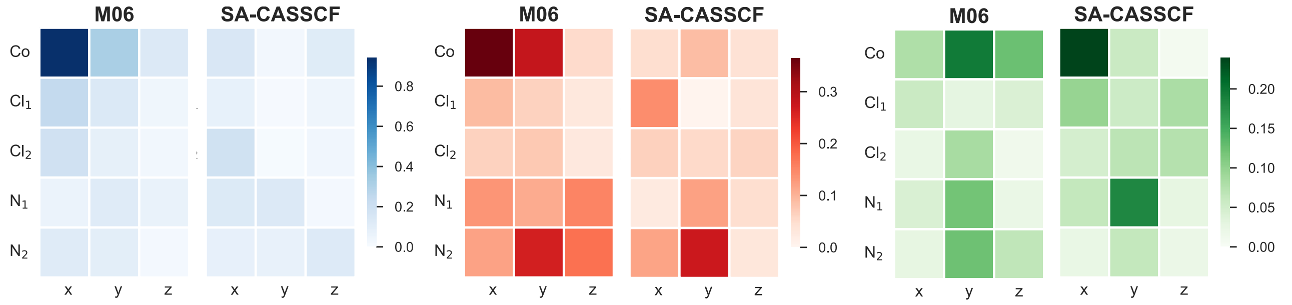

Figure S14. Non-adiabatic couplings between the ground and first three excited states of Co(II)(sp)Cl_2 calculated with M06 and SA-CASSCF.

S5.2. Ni(sp)Cl_2

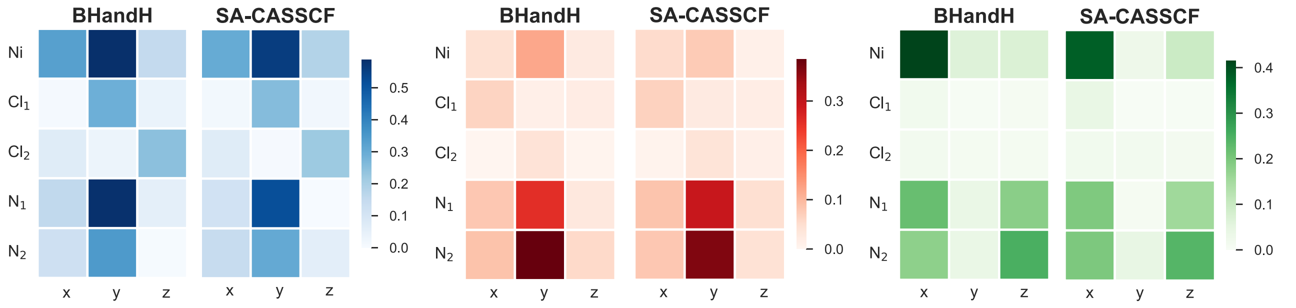

Figure S15. Non-adiabatic couplings between ground and first three excited states of Ni(II)(sp)Cl_2 calculated with BHandH and SA-CASSCF for atoms around Ni(II): (a) ground and first excited state; (b) ground and second excited state; (c) ground and third excited state.

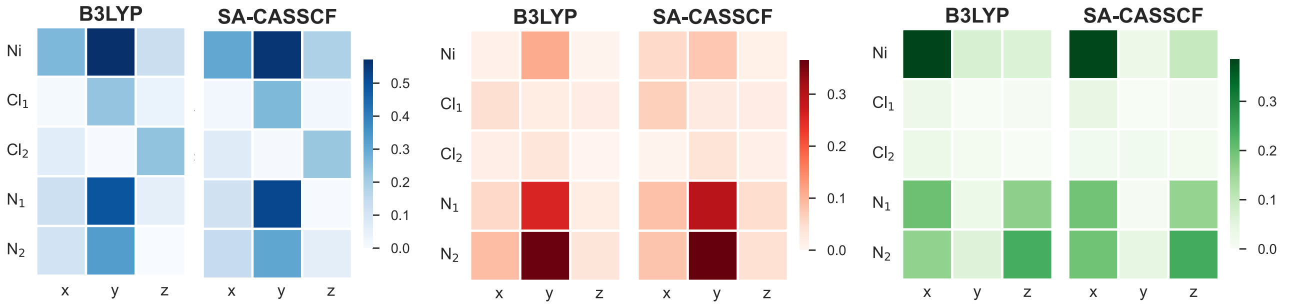

Figure S16. Non-adiabatic couplings between the ground and first three excited states of Ni(II)(sp)Cl_2 calculated with B3LYP and SA-CASSCF.

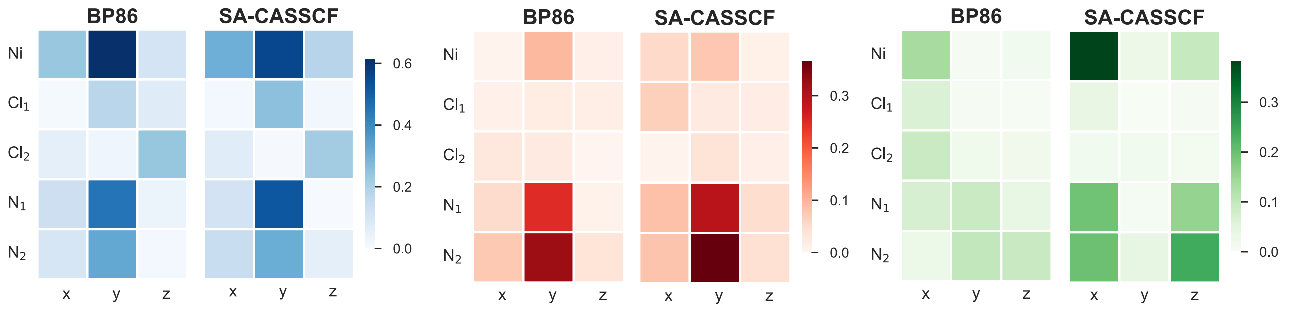

Figure S17. Non-adiabatic couplings between the ground and first three excited states of Ni(II)(sp)Cl_2 calculated with BP86 and SA-CASSCF.

S5.3. NAC Analysis

REF: Reference value is calculated with SA-CASCCF/ANO-RCC-VTZP (ANO-RCC-MB for C and H atoms)
MAE: Mean absolute error
MAX: Maximum absolute error
RMSE: Root-mean-square error
L: Largest element of the reference NACs for the nth excitation

Table S11. Comparison of non-adiabatic couplings between the ground and first three excited states of Co(II)(sp)Cl_2 calculated with TDDFT and SA-CASSCF.

		BHandH			B3LYP		REF
#.	MAE	MAX	RMSE	MAE	MAX	RMSE	L
1	0.0075	0.127	0.0182	0.0085	0.105	0.0185	0.187
2	0.0035	0.047	0.0092	0.0038	0.049	0.0089	0.272
3	0.0095	0.151	0.0264	0.0101	0.138	0.0265	0.239
		BP86			M06		Ref
#.	MAE	MAX	RMSE	MAE	MAX	RMSE	L
1	0.0087	0.123	0.0198	0.0184	0.792	0.0756	0.187
2	0.0102	0.269	0.0309	0.0108	0.315	0.0375	0.272
3	0.0074	0.147	0.0189	0.0081	0.160	0.0240	0.239

Table S12. Comparison of non-adiabatic couplings between the ground and first three excited states of Ni(II)(sp)Cl_2 calculated with TDDFT and SA-CASSCF.

		BHandH			B3LYP		REF
#.	MAE	MAX	RMSE	MAE	MAX	RMSE	L
1	0.0054	0.065	0.0126	0.0051	0.058	0.0113	0.556
2	0.0023	0.043	0.0060	0.0031	0.042	0.0075	0.364
3	0.0027	0.036	0.0071	0.0028	0.043	0.0069	0.381
		BP86		-	-	-	REF
#.	MAE	MAX	RMSE	-	-	-	L
1	0.0073	0.081	0.0164	-	-	-	0.556
2	0.0042	0.055	0.0103	-	-	-	0.364
3	0.0108	0.245	0.0347	-	-	-	0.381

S6. Coordinates

Co(sp)Cl_2 xyz coordinates optimized BP86-D3BJ-CPCM/def2-TZVP with Orca

46
Coordinates from ORCA-job ./1547094 E -3000.823867165991
Co 6.99491453281983 2.24406221066966 4.02630250572301
Cl 6.70330783203854 0.29400169709201 5.08860781728658
Cl 7.28427211379117 3.93778459369101 5.45458497553286
N 8.24010279292044 1.89949072769322 2.42310173040006
N 5.43130762330139 2.68527761702432 2.77649028758049
C 9.59073750876111 1.46472620779643 2.88040007767439
C 10.30409641634177 2.56372948955086 3.65580591234117
C 10.41065919317730 3.84083004341960 2.81813409137016
C 9.03099154449875 4.25997785472419 2.30159332955260
C 8.34849295161739 3.11805698078779 1.55347478974527
C 6.98210210742848 3.48611212648167 0.94320256314631
C 6.51398948029109 2.32706054061533 0.06237132877358
C 6.27787853565226 1.13981052424501 1.00045740941212
C 7.60621433458457 0.77124078290542 1.67338627439194
C 5.12817851066320 1.44347467077859 1.97997291889873
C 3.76829162931718 1.50879295036679 1.26503282157835
C 2.62986813668139 1.85651701959809 2.22593310638952
C 2.94856108618489 3.16414956746127 2.95199492540423
C 4.29142131964243 3.04865252030990 3.66996187820806
C 5.86691717652113 3.84279581518216 1.93933659779788
H 9.44743583103578 0.56682090317574 3.49698102891343
H 10.18080855801033 1.17752772470641 1.98812803790420
H 11.30037746469425 2.19754315369848 3.94420014010886
H 9.75064291118618 2.77268922542419 4.58535480861138
H 11.08050473888631 3.65934009510743 1.95986146624239
H 10.85732070580217 4.65258191168023 3.41033504355401
H 9.11642760270600 5.11187566489389 1.60991937149900
H 8.40600987895263 4.58499067443670 3.14768515682741
H 9.00168889180989 2.83552699443736 0.70086745678337
H 7.15207454261539 4.38633958327442 0.33275837935557
H 7.28733659538781 2.07526849667293 -0.67889798541524
H 5.60428149741039 2.59380437461121 -0.49212985383906
H 5.96999705070729 0.25317420411060 0.42507748917769
H 7.47686341804145 -0.05589476407808 2.38481209279967
H 8.31246323224256 0.44213718085385 0.88823432209172
H 5.07901291336170 0.63536735639448 2.72861993441078
H 3.59658505568220 0.53105505815216 0.78973739984077
H 3.79575990840834 2.25479089046548 0.45628943686213
H 2.50740090377449 1.04730244487819 2.96627991495633
H 1.68065091780832 1.93548364129135 1.67568982671273
H 2.17749561757374 3.40401331404449 3.69907312283527
H 2.96707493522768 3.99933958743695 2.23334310146731
H 4.22951563387586 2.25729601944403 4.43379888823564
H 4.56865564251477 3.98016024267931 4.18040951858740
H 5.01382989443260 4.23846473766678 1.36352237746082
H 6.18467183161722 4.63078434414798 2.63658818280902

Ni(sp)Cl$_2$ xyz coordinates optimized BP86-D3BJ-CPCM/def2-TZVP with Orca

46
Coordinates from ORCA-job ./1575556 E -3126.391533153851
Ni 7.00931485390759 2.28716670001971 4.03639137631787
Cl 6.71655246651106 0.21645203267263 4.87082115106370
Cl 7.19253537463040 4.12869457248019 5.30175142403506
N 8.20857916716811 1.89843429345315 2.42596679904731
N 5.45828402580587 2.67870469047847 2.77204097859377
C 9.54509560849001 1.46294259012908 2.92626177573253
C 10.25471138073396 2.56768248600956 3.69629333596127
C 10.40224969590729 3.82339083892752 2.83336039150064
C 9.04202969822863 4.25647817164119 2.27911947576048
C 8.34359943906593 3.11149220120820 1.55158516729836
C 6.98419970117315 3.48966939682434 0.93344220754402
C 6.51141945097433 2.33581716871398 0.04757110497657
C 6.27963964758585 1.14296553683758 0.98018912043956
C 7.60546429298595 0.77455759434411 1.65166407653581
C 5.14393981813882 1.44249593194074 1.97401802622676
C 3.77588008637925 1.51907618400015 1.27461392402065
C 2.64953881633852 1.84953914219374 2.25461129279115
C 2.97747008738055 3.14617498012383 2.99429135242037
C 4.33241205785945 3.02782123981649 3.68975612628662
C 5.87262545339284 3.84044266484799 1.93379548214214
H 9.37788656973382 0.57608095753627 3.55186961568862
H 10.15122511593420 1.15745464178756 2.05153125794144
H 11.23765716247431 2.19098313631359 4.01530357683689
H 9.68647407253876 2.80473736161278 4.61035836212603
H 11.08771465845713 3.60918334870016 1.99513067402002
H 10.85133555593929 4.64028266476372 3.41657263936425
H 9.15947956623061 5.08249986759869 1.56134470152929
H 8.40890472775527 4.62108054007908 3.10089019879428
H 8.99562088668988 2.80394995875470 0.70700371356922
H 7.15795821894673 4.39248788065211 0.32815735788046
H 7.28072076114341 2.08785057878167 -0.69911846243744
H 5.59900348717027 2.60710842025188 -0.50048883269864
H 5.96670702166156 0.25983257272563 0.40226600137984
H 7.48084985512036 -0.06478866460493 2.34686117741141
H 8.32607399341021 0.47694712664715 0.86725036409570
H 5.10245981050645 0.63314893269764 2.71925574585225
H 3.60186296895349 0.54766201200440 0.78742908764617
H 3.79227705242977 2.27608626865677 0.47595101415557
H 2.53865364591505 1.02913429628648 2.98415403159892
H 1.69265779920500 1.93596278227651 1.71882997682828
H 2.22104246039780 3.37238325280134 3.76037232353575
H 2.98046648910183 3.99332026705759 2.28961714426672
H 4.28744682968110 2.22608387797475 4.44343971831607
H 4.61411941604114 3.95428306930081 4.20494589095729
H 5.00920499329402 4.21891076430716 1.36184521135294
H 6.18384675861024 4.63166266837362 2.62836692129382

$Zn(sp)Cl_2$ xyz coordinates optimized BP86-D3BJ-CPCM/def2-TZVP with Orca

46
Coordinates from ORCA-job ./1547012 E -3397.471564112006
Zn 0.20951328801406 -0.10890216115077 0.97077548287767
Cl -0.14247522293437 -2.11489760718642 1.95174339162007
Cl 0.47917075142205 1.64711068412979 2.35030112310752
N 1.45479917802831 -0.48083470109146 -0.69437334812239
N -1.35773968050643 0.33719834879067 -0.36430632301056
C 2.80978122216301 -0.91659427426723 -0.25022670251140
C 3.54217460338996 0.18811111671449 0.49958829145693
C 3.64048775779211 1.45750349869013 -0.35100821770260
C 2.25621465960435 1.87106039784801 -0.85994151280445
C 1.56798775303315 0.72031562894234 -1.58748385304569
C 0.20608657072001 1.08546918047574 -2.20813115370356
C -0.26699139389147 -0.08864929888836 -3.06568295918427
C -0.51482623899871 -1.25277822685190 -2.10200392240186
C 0.81087289615103 -1.61739969009043 -1.42117698245451
C -1.66737419327122 -0.91813884410517 -1.13517343145588
C -3.02017277400515 -0.86054701116619 -1.86312742502324
C -4.16464927214199 -0.47500293841755 -0.92396959646040
C -3.83877149733939 0.84876452021432 -0.23028972270451
C -2.50667021591834 0.73480564831673 0.50485172721466
C -0.90954882513805 1.47241303698411 -1.22467117852359
H 2.67267640002061 -1.80531943050993 0.38149805481660
H 3.38368206596333 -1.22045132360512 -1.14709512555511
H 4.54151109560267 -0.17917933173145 0.77552982150241
H 3.00860593887382 0.40861605041102 1.43827077860731
H 4.30307264644813 1.26869021556324 -1.21313427581076
H 4.09234033889193 2.27432752704832 0.23021999109589
H 2.33541687123461 2.71662246952931 -1.55992969768040
H 1.63669684563984 2.20445878562502 -0.01290358390265
H 2.22043728514235 0.41773420144555 -2.43409341576822
H 0.38600209224315 1.97096022311173 -2.83726798517272
H 0.50765278505069 -0.36201272272309 -3.79806205070105
H -1.17247309276757 0.17231235151488 -3.62972703066922
H -0.82785828354550 -2.14962407023703 -2.65872761623782
H 0.67421018106860 -2.42917053687522 -0.69350901390853
H 1.51286460982651 -1.96764998902467 -2.20098610294294
H -1.72662122675468 -1.71107319651494 -0.37062977206633
H -3.19824997327448 -1.84925437925902 -2.31301208589092
H -2.97948779484096 -0.13821414398489 -2.69279214879391
H -4.30253744910006 -1.26196250149924 -0.16243311434213
H -5.10798674559790 -0.40204847035557 -1.48506636208841
H -4.61570713606069 1.11709559475840 0.50097955596981
H -3.80443287939292 1.66378242212526 -0.97110980395163
H -2.59119727392156 -0.03664189848063 1.28729522465077
H -2.22417524637777 1.67434361103609 0.99837417991745
H -1.75524543084545 1.86572748903347 -1.81230710480081
H -0.58395125579960 2.27184900610761 -0.54387507684456

References

[1] K. Wiberg, W. Bailey, *Journal of Molecular Structure* **2000**, *556*, 239.

[2] P. Pracht, F. Bohle, S. Grimme, *Phys. Chem. Chem. Phys.* **2020**, *22*, 7169.

[3] AMS 2025.1, SCM, Theoretical Chemistry, Vrije Universiteit, Amsterdam, The Netherlands, see https://www.scm.com/ .

[4] S. Grimme, C. Bannwarth, P. Shushkov, *Journal of Chemical Theory and Computation* **2017**, *13*, 1989, pMID: 28418654.

[5] A. D. Becke, *Phys. Rev. A* **1988**, *38*, 3098.

[6] J. P. Perdew, *Phys. Rev. B* **1986**, *33*, 8822.

[7] S. Grimme, J. Antony, S. Ehrlich, H. Krieg, *The Journal of chemical physics* **2010**, *132*.

[8] S. Grimme, S. Ehrlich, L. Goerigk, *Journal of computational chemistry* **2011**, *32*, 1456.

[9] V. Barone, M. Cossi, *The Journal of Physical Chemistry A* **1998**, *102*, 1995.

[10] F. Neese, *WIRES Comput. Molec. Sci.* **2012**, *2*, 73.

[11] F. Weigend, R. Ahlrichs, *Phys. Chem. Chem. Phys.* **2005**, *7*, 3297.

[12] F. Weigend, *Journal of Computational Chemistry* **2008**, *29*, 167.

[13] G. Li Manni, I. Fdez. Galván, A. Alavi, F. Aleotti, F. Aquilante, J. Autschbach, D. Avagliano, A. Baiardi, J. J. Bao, S. Battaglia, L. Birnoschi, A. Blanco-González, S. I. Bokarev, R. Broer, R. Cacciari, P. B. Calio, R. K. Carlson, R. Carvalho Couto, L. Cerdán, L. F. Chibotaru, N. F. Chilton, J. R. Church, I. Conti, S. Coriani, J. Cuéllar-Zuquin, R. E. Daoud, N. Dattani, P. Decleva, C. de Graaf, M. G. Delcey, L. De Vico, W. Dobrautz, S. S. Dong, R. Feng, N. Ferré, M. Filatov(Gulak), L. Gagliardi, M. Garavelli, L. González, Y. Guan, M. Guo, M. R. Hennefarth, M. R. Hermes, C. E. Hoyer, M. Huix-Rotllant, V. K. Jaiswal, A. Kaiser, D. S. Kaliakin, M. Khamesian, D. S. King, V. Kochetov, M. Kronicki, A. A. Kumaar, E. D. Larsson, S. Lehtola, M.-B. Lepetit, H. Lischka, P. López Ríos, M. Lundberg, D. Ma, S. Mai, P. Marquetand, I. C. D. Merritt, F. Montorsi, M. Mörchen, A. Nenov, V. H. A. Nguyen, Y. Nishimoto, M. S. Oakley, M. Olivucci, M. Oppel, D. Padula, R. Pandharkar, Q. M. Phung, F. Plasser, G. Raggi, E. Rebolini, M. Reiher, I. Rivalta, D. Roca-Sanjuán, T. Romig, A. A. Safari, A. Sánchez-Mansilla, A. M. Sand, I. Schapiro, T. R. Scott, J. Segarra-Martí, F. Segatta, D.-C. Sergentu, P. Sharma, R. Shepard, Y. Shu, J. K. Staab, T. P. Straatsma, L. K. Sørensen, B. N. C. Tenorio, D. G. Truhlar, L. Ungur, M. Vacher, V. Veryazov, T. A. VoSS, O. Weser, D. Wu, X. Yang, D. Yarkony, C. Zhou, J. P. Zobel, R. Lindh, *Journal of Chemical Theory and Computation* **2023**, *19*, 6933, pMID: 37216210.

[14] B. O. Roos, R. Lindh, P.-A. Malmqvist, V. Veryazov, P.-O. Widmark, *The Journal of Physical Chemistry A* **2005**, *109*, 6575, pMID: 16834004.

[15] B. O. Roos, R. Lindh, P.-A. Malmqvist, V. Veryazov, P.-O. Widmark, *The Journal of Physical Chemistry A* **2004**, *108*, 2851.

[16] J. Shen, C. Zhu, S. Reiling, R. Vaz, *Spectrochimica Acta Part A: Molecular and Biomolecular Spectroscopy* **2010**, *76*, 418.

[17] Y. He, *Enhanced VCD in Transition Metal Complexes and Metalloproteins*, Ph.D. thesis, Syracuse University **2005**.

[18] J. P. Perdew, *Phys. Rev. B* **1986**, *33*, 8822.

[19] P. J. Stephens, F. J. Devlin, C. F. Chabalowski, M. J. Frisch, *The Journal of Physical Chemistry* **1994**, *98*, 11623.

[20] M. Bühl, H. Kabrede, *Journal of Chemical Theory and Computation* **2006**, *2*, 1282, pMID: 26626836.

[21] K. P. Jensen, B. O. Roos, U. Ryde, *The Journal of Chemical Physics* **2007**, *126*, 014103.

[22] Y. Minenkov, A. Singstad, G. Occhipinti, V. R. Jensen, *Dalton Trans.* **2012**, *41*, 5526.

[23] C. Zhao, R. Wu, S. Zhang, X. Hong, *The Journal of Physical Chemistry A* **2023**, *127*, 6791, pMID: 37530446.

[24] M. Ernzerhof, G. E. Scuseria, *The Journal of Chemical Physics* **1999**, *110*, 5029.

[25] M. A. J. Koenis, Y. Xia, S. R. Domingos, L. Visscher, W. J. Buma, V. P. Nicu, *Chem. Sci.* **2019**, *10*, 7680.

[26] E. J. Baerends, N. F. Aguirre, N. D. Austin, J. Autschbach, F. M. Bickelhaupt, R. Bulo, C. Cappelli, A. C. T. van Duin, F. Egidi, C. Fonseca Guerra, A. Forster, M. Franchini, T. P. M. Goumans, T. Heine, M. Hellstrom, C. R. Jacob, L. Jensen, M. Krykunov, E. van Lenthe, A. Michalak, M. M. Mitoraj, J. Neugebauer, V. P. Nicu, P. Philipsen, H. Ramanantoanina, R. Ruger, G. Schreckenbach, M. Stener, M. Swart, J. M. Thijssen, T. Trnka, L. Visscher, A. Yakovlev, S. van Gisbergen, *Journal of Chemical Physics* **2025**, *162*, 162501.
